# Non-Coding RNAs Are Brokers in Breast Cancer Interactome Networks and Add Discrimination Power between Subtypes

**DOI:** 10.3390/jcm11082103

**Published:** 2022-04-09

**Authors:** Ankush Sharma, Enrico Capobianco

**Affiliations:** 1 Department of Biosciences, University of Oslo, 0315 Oslo, Norway; ankush.sharma@medisin.uio.no; 2 Department of Bioinformatics, University of Oslo, 0315 Oslo, Norway; 3 Institute of Data Science and Computing, University of Miami, Coral Gables, FL 33146, USA

**Keywords:** regulatory networks, breast cancer subtypes, non-coding RNAs

## Abstract

Despite the power of high-throughput genomics, most non-coding RNA (ncRNA) biotypes remain hard to identify, characterize, and validate. This is a clear indication that intensive next-generation sequencing research has led to great efficiency and accuracy in detecting ncRNAs, but not in their functionalization. Computational scientists continue to support the discovery process by spotting significant data features (expression or mutational profiles), elucidating phenotype uncertainty, and delineating complex regulation landscapes for biological pathways and pathophysiological processes. With reference to transcriptome regulation dynamics in cancer, this work introduces a novel network-driven inference approach designed to reveal the potential role of computationally identified ncRNAs in discriminating between breast cancer (BC) subtypes beyond the traditional gene expression signatures. As heterogeneity cast in the subtypes is a characteristic of most cancers, the proposed approach is generalizable beyond BC. Expression profiles of a wide transcriptome spectrum were obtained for a number of BC patients (and controls) listed in TCGA and processed with RNA-Seq. The well-known PAM50 subtype signature was available for the samples and used to move from differentially expressed transcript profiles to subtype-specific biclusters associating gene patterns with patients. Co-expressed gene networks were then generated and annotations were provided, focusing on the biclusters with basal and luminal signatures. These were used to build template maps, i.e., networks in which to embed the ncRNAs and contextually functionalize them based on their interactors. This inference approach is able to assess the influence of ncRNAs at the level of BC subtype. Network topology was considered through the brokerage measure to account for disruptiveness effects induced by the removal of nodes corresponding to ncRNAs. Equivalently, it is shown that ncRNAs can act as brokers of network interactome dynamics, and removing them allows the refinement of subtype-related characteristics previously obtained by gene signatures only. The results of the study elucidate the role of pseudogenes in two major BC subtypes, considering the contextual annotations. Put into a wider perspective, ncRNA brokers may help predictive functionalization studies targeted to new disease phenotypes, for instance those linked to the tumor microenvironment or metabolism, or those specifically involving metastasis. Overall, the approach may represent an in silico prioritization strategy toward the systems identification of new diagnostic and prognostic biomarkers.

## 1. Introduction

The advent of high-throughput genomics has provided lots of examples in which non-coding RNA (ncRNA) potentially (co-)regulates physiological programs in developmental and disease contexts [1], although the specific functions of ncRNA remain largely unknown [1,2]. In general, ncRNAs are key players in epigenetic regulation, such as chromatin modification and remodeling, methylation, cellular reprogramming, etc. [3]. Specifically, in cancer, the disruption of normal biological functionality at various levels (transcriptional, post transcriptional, post translational, etc.) [4,5] is a complex process with increasingly evident but yet-to-be defined roles played by a variety of ncRNA biotypes. Deciphering such complexity, usually summarized into high-throughput driven genomic or transcriptomic signatures, mutational profiles, risk factors, etc., typically requires two types of support: (1) computational support via tools designed to discover correlative or even causal associations between the identified bioentities; (2) qualitative support via development and/or update of accurate bio-annotation repositories with increasingly integrated contents.

Going into more detail regarding the computational support, the configuration of network maps connecting ncRNAs with their targets (mRNA transcripts) (see [6,7], among others) offers a good example of inference methodologies aimed at facilitating the assessment of complex regulations of the functional mechanisms induced by the disease. Networks are ideal mathematical frameworks for conducting such complex inferences [8,9]. Because of the influence of ncRNAs on the expression levels of gene targets, connecting their joint profiles via networks allows the measure of both structural and dynamic features at large modular scale [10]. As ncRNAs can be considered as flexible modular scaffolds that contain discrete domains interacting with proteins [11], network modularity likely emphasizes the synergistic functionality between regulatory proteins. Ultimately, ncRNAs are considered possible drivers of multiple cancer hallmarks responsible for tumor initiation, growth, and progression. In principle, network maps including RNA structures might lead to the identification of more robust cancer signatures [12,13] and, in turn, more effective validations.

For the second type of support, the role of integrating contextual information into biological networks is multilevel, i.e., defined through structure, topology, interaction dynamics and functions. The convolution induced by many possible regulatory patterns suggests to reduce redundancy and prioritize the ncRNA functional roles. In other terms, a goal is to identify for each ncRNA an informative context that the network can help analyze and infer from. The focus is traditionally on ncRNA transcription, in particular establishing significance of *cis-* (from the allele of transcription) and *trans*- (not restricted to neighbor dependence) acting associations. However, most ncRNAs are not co-expressed with neighbor coding genes, which diminishes the relevance of the proximity criterion. In practice, identifying the putative functional role of ncRNAs requires perturbation experiments and, more realistically, a systems approach to characterize multiple ncRNA co-regulation dynamics. This is also an experimental challenge due to phenotypic uncertainty.

As an alternative derived from the convergence of the two described supports, ncRNA functions and processes are typically inferred in silico through the ‘guilt-by-association’ principle, coming with the inherent limitation of bringing just correlative (non-causative) evidence potentially akin to trans-regulatory models (see [14]). In general, high-degree nodes (hubs) have been associated with essential genes [15,16], while nodes associated to topological centrality have been found negatively correlated with the evolutionary rate [17]. Both types of nodes are considered ‘connectors’ as they bridge between network nodes, shape the local modularity, establish network gateways that consolidate communities, contextualize the dynamics of interconnected nodes, and determine the level of coordination underlying multiple functional components. Intuitively, the collective properties emerging from synergistic bio-entities, when functionally contextualized, make the network topology highly instrumental to the discovery of cancer phenotypes.

A demonstration of this hypothesis is proposed in this work. Breast cancer (BC) data from The Cancer Genome Atlas (TGCA) samples were profiled with RNA-Seq considering the PAM50 gene signature (a 50-gene signature that identifies the biological subtype of a given sample). As the subtypes (luminal A, luminal B, basal-like, Her2-enriched and normal-like [18]) depend on unsupervised statistical methods, some uncertainty remains regarding the biological relevance of the classified genes in terms of their inherent predictive power toward the complex regulation of expression levels subject to the influence of such molecular subtypes [19]. Because of the current limited knowledge about the potential role of ncRNAs as potential regulators of the dynamics underlying cancer phenotypes, the significance of the proposed computational approach is to show that BC- subtype patient stratifications may indeed be co-determined by latencies and synergies centered on the potential regulation of expression dynamics by ncRNA and elucidated here for the first time by network topology. Specifically, by showing that ncRNAs may act as network brokers, we propose an in silico perturbation strategy through which inference can be conducted and further validated.

## 2. Materials and Methods

### 2.1. Inference Approach

**Figure 1:**
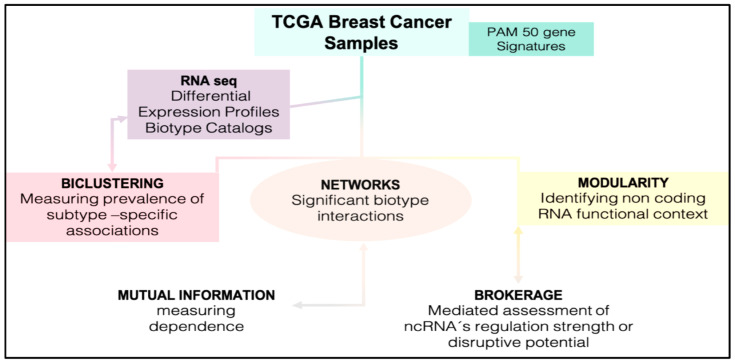
Workflow.

A four-step inference approach is displayed in Figure 1 (activity workflow). The steps are aimed to identify significant influences of ncRNAs measure at network scale. First, the biotypes were profiled from RNA-Seq BC samples of the TGCA repository. Second, transcriptome-driven biclusters were computed from differentially expressed genes (DEG) with sample subtype-specific (PAM50) signature. Third, the most representative basal and luminal biclusters were selected to build the associated co-expressed networks. Fourth, the significantly measured ncRNAs were mapped onto such networks and modularity was computed to generate functional contexts. The regulatory potential of these ncRNAs was quantitatively assessed by brokerage.

### 2.2. Dataset 

TCGA BC data are randomly sampled (106 normal, 124 tumor) to form a reference paired-end RNA-Seq baseline. The tissue type for all samples is infiltrating ductal carcinoma from female patients (median age: 57 years for normal samples; 52.5 years for tumor samples). The sequencing is performed on Illumina Hi-Seq 2000 (San Diego, CA, USA), with each sample containing approximately 100 mL reads. In terms of quality control, the only retained reads have a mapping quality (MAQ) score ≥20 and a mapping rate >90% against repeat masked human transcriptome (hg19). Fragments per kilobase per million reads (FPKM) are computed via standard mapping and quantification (Tophat v.1-08, Cufflinks 2). Normalization (quantile and log2 transform) and differential expression (DE) analysis (via Student’s *t*-test) were computed with GeneSpring V13 (Agilent) Of 56,471 identifications including protein coding genes, pseudogenes, antisense and lncRNAs, 16,051 are from tumor samples, with 15,466 biotypes passing the cut-off (coeff. variation > 0 across samples) (See also Supplementary Materials File S1).

### 2.3. Biclustering

From the measured profiles of DE genes (DEGs), a few basic structures were computed:

(a) A normalized matrix V = (X,Y) describing the value of gene *i* in sample *j* for all (i,j), given X genes: X = [x_1_, x_2_, …, x_N_] and Y samples: Y = [y_1_,y_2_,…,y_M_].
(b) A bicluster B = (I,J) as the *n x m* sub-matrix of V obtained by matching a subset of genes I = (i_1_, i_2_, …, i_n_) with a subset of samples J = (j_1_, j_2_, …, j_m_).
(c) The target biclusters forming a vector B^k^ = (B_1_, B_2_, …, B_k_) with each B representing a different combination of BC subtypes as per the PAM50 signature. Thus, non-disjoint subsets were found (i.e., each B includes subtype-specific fractions of genes).
(d) The signature biclusters, i.e., those selected in B^k^ algorithmically by the Iterative Signature Algorithm [20,21,22] (R-Bioconductor ISA2 package V0.3.5) and with at least one prevalent subtype (>50% of genes present in the set).

### 2.4. Networks

The DEGs extracted from the signature biclusters identified were used to build gene co-expression interactome networks, i.e., template networks (TNs). Emphasis was assigned especially to two subtypes, basal and luminal. The related TN motivated further in-depth topological analysis to guide biological annotation of the profiled genes at a modular scale and to characterize the ncRNA through the established functional contexts. In principle, the complexity of regulatory networks associated to ncRNAs would refer to multiple nodes possibly regulating proteins and complexes. By targeting the annotated functional contexts associated to the ncRNAs, an inherent reduction of redundancy and complexity is obtained due to a shift from single to interconnected ncRNA interacting genes. Equivalently, the genes interacting with the target ncRNAs are considered functionally active and inform on potential regulation dynamics.

The TN convolution induced by the ncRNAs reflects the connectivity patterns between them and the genes, and these relationships require further assessment for significance. The choice of the metric is key for determining how the ncRNAs engage into the functional contexts. Both correlation (COR) and mutual information (MI) metrics were considered. While MI revealed more robust and yielded consistency of significant links between ncRNA and DEG nodes tested against random effects, COR showed less consistent interactomes under random permutations and less significant ncRNA-mRNA associations under network randomization. The MI metric was thus preferred and converted into a corresponding distance function, *d =* 1 − *MI*, to accommodate all the interactions between ncRNAs and genes measured with high confidence. Frequency plots were generated to assess network rewiring (scrambling or randomizing 100 times, iterating 1000 times) and inform on stability (checked via number of persistent network links). Despite MI being expected to be more stable with regards to consistency of links against randomization, sensitivity to the ncRNAs presence and specific subtype influence were observed with both metrics, implying that different levels of regulative convolution can be present depending on the chosen metric (see Supplementary Materials File S2).

### 2.5. Modularity

The tool to measure modularity, ModuLand, V1.3 searches for overlapping modules by identifying the hills of an influence function-derived centrality measure determining a community landscape [23,24]. The methodology leads to first defining an influence function-based centrality measure whose hills represent overlapping modules, then constructing a community landscape and finding the hills before finally identifying a hierarchy of networks.

### 2.6. Role of Brokerage

Our network inference strategy leverages the topological relevance of a network measure known as ‘brokerage’ [25,26]. This property considers nodes as brokers because they connect nodes that would be otherwise disconnected. Intuitively, removing these brokers can cause network disruption to more-or-less localized extent. In terms of functionalization, the approach allows screening and ranking of ncRNAs and DEG by brokerage values normalized at network scale but contextualized as per the identified modules. This way, by spotting critical network nodes, i.e., the ncRNAs with high-brokerage values, and by associating them with the gene interactors across a variety of functionally annotated contexts, the ncRNA brokers inform about potential disruptive effects that become measurable once they are removed. Because our conjecture is that some ncRNAs might contribute to establishing subtype signatures, measuring the effects of disruptiveness may help explain the limited capacity of gene signatures alone to identify subtypes.

Specifically, brokerage assigns quantitative significance to nodes proportionally to their local neighborhood interconnectedness. The consideration of the functional contexts allows a qualitative assessment of such significance. This property can thus reinforce the meaning of density of a cluster of nodes, but can also be seen as an edge property when looking at the connection between clusters (bridging), with the result of augmenting the network efficiency relative to the flow of information [27]. Note that bridgeness differentiates the edges according to their structural relevance and is not necessarily associated with the well-known betweenness centrality property [28]. In fact, a high-betweenness edge is a bottleneck for which removal interrupts network communication among nodes along the shortest paths, as per the definition of betweenness centrality, but without implying a bridge (a cut-edge). The latter may have not high betweenness and still increase the number of connected components, thus representing a type of network vulnerability (in parallel, an articulation point or cut-vertex can be considered too [29]).

In general, the disruptive potential of nodes over the network configuration is measurable by how disconnected this may become after removing such nodes. However, only a few measures are informative. At a node *i*, the clustering coefficient *C_i_* is the probability of two node neighbors of *i* being connected by a link and the effective size *S_i_* of node *i* and indicates the extent to which each of its neighbors is redundant (this measure is inversely proportional to *C_i_*). The local efficiency *E_i_* is a generalization of *C_i_* referred to the subgraph *G_i_* (the average of the inverse of the distances between the nodes of the graph, determined by where the links are located). Compared to *C_i_*, the role of *E_i_* informs about the topological variation of *G_i_*: having small or large *E_i_* implies that the node *i* is a broker depending on whether its removal would or would not disconnect its neighbors. For our purposes, the local brokerage *B_i_* is proportional to *E_i_* according to *B_i_* = *k_i_* − (*k_i_* − 1)*E_i_*. Importantly, a highly brokered network is likely more vulnerable to attack and dependent on relatively few nodes to remain fully functional.

### 2.7. Pseudogenes

Of the thousands of ENCODE-annotated human pseudogenes, a great majority was found transcriptionally active [30]. Pseudogenes share high sequence homology to their functional counterparts, but in most cases are unable to produce functional proteins. There is an abundance of them; about 10% of human genes have a pseudogene counterpart and genes often have multiple counterparts. Although they do not generate functional protein products, pseudogenes may act as regulatory RNAs and affect the expression of coding genes through multiple mechanisms.

Pseudogenes have also been shown to be relevant to disease, including cancer [31]; they can regulate the expression of their functional counterparts and impact tumor development. Differentially expressed pseudogenes were systematically revealed in the transcriptional landscape of human cancers [32], and the wide-spectrum RNA-seq transcriptomes obtained from TCGA has characterized the pseudogene expression profiles of cancer patients. This type of evidence demonstrates the predictive power of pseudogenes for biomarker discovery is comparable to that of mRNA. This goes together with the ability of jointly capturing clinically relevant information and serve as independent validation instruments for the classification of tumor subtypes [33].

Finally, it is very relevant to our work hypothesis that pseudogene subtypes have been found highly concordant with the well-established PAM50 molecular subtypes and also correlated with the mutation status of key cancer genes [34]. In a TCGA pan-cancer study, about one-third of the most significantly enriched 100 genes were pseudogenes, although none of their functional counterparts were in the list. This is something we also verified in our smaller study, indicating once again that the expressed pseudogenes might, in principle, discriminate tumor types at much larger scale than their functional counterparts (target genes) [35].

## 3. Results

### 3.1. Biclustering

Cross-analysis of normal and tumor samples has delivered a profile of 2344 significant DEGs (*p*-values ≤ 0.001, fold-change (FC) ≥ 2) and an associated profile of DE non-protein coding biotypes including 735 pseudogenes, 183 antisense, 130 lincRNAs, 35 processed transcripts and 58 repeats. At the gene level, in order to reach a suitable data partitioning, 326 biclusters were identified by ISA with bicluster size constrained to 10 genes and 7 samples. Other algorithmic constraints (i.e., conditional optimization and robustness thresholds > 100) were applied separately to normal (6) and tumor (13) samples, leaving the 13 most significant biclusters. The subtype classification of tumor biclusters is shown in Table 1 (see also Supplementary Materials File S3).

### 3.2. Network Maps

**Figure 2:**
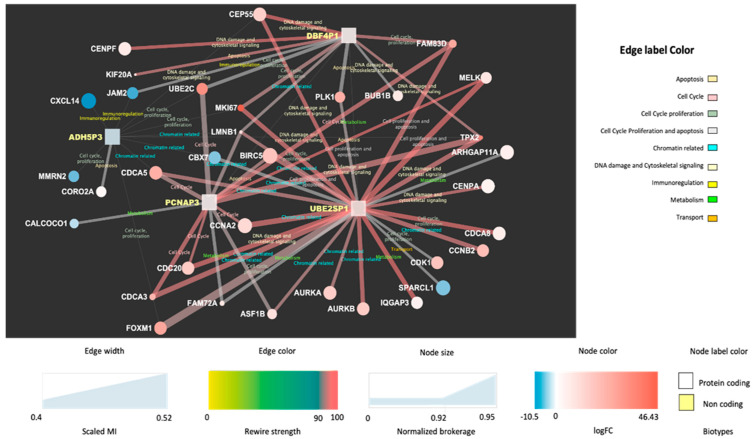
Basal map with top brokerage ncRNAs: DBF4P, ADH5P3, PCNAP3, UBE2SP1.

**Figure 3:**
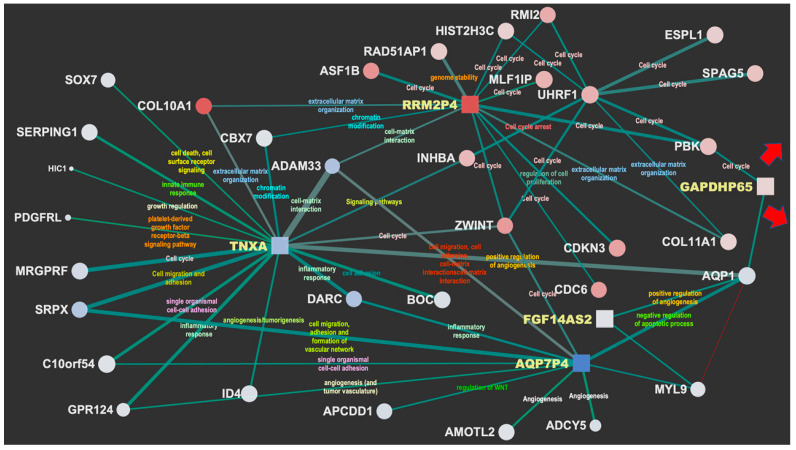

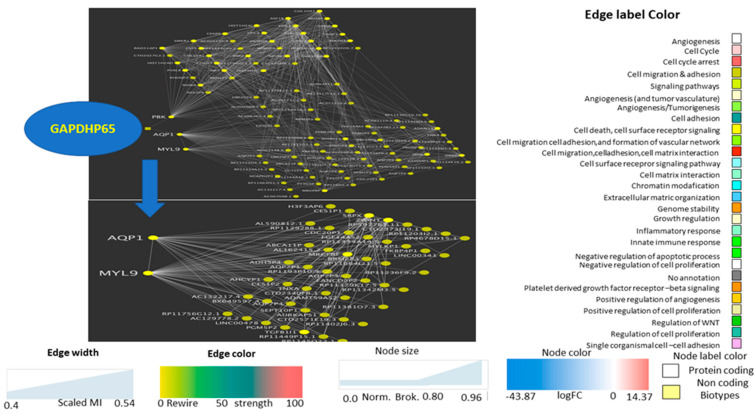
**Top** panel. Luminal map and top brokerage ncRNAs: TNXA, AQP7P4, FGF14AS2, GAPDHP65, RRM2P4. Red arrows indicate graph extension. **Bottom** panel. Annotations and linked GAPDHP65 subnetwork.

Figure 2 and Figure 3 display the network interactions maps obtained from the selected reference biclusters with basal and luminal signatures, respectively. The maps are integrative views of interaction landscapes between protein coding genes and ncRNAs. This match is obtained though sequential streps. First, two gene co-expression networks were derived from the genes annotated for biological processes within the reference biaclusters. Then, the ncRNAs were mapped onto the networks (visible as square nodes) with size depending on the computed normalized brokerage values and color reflecting the measured logFC. The edges carry information on the scaled MI (measured by the edge width) and the rewiring strength (edge color) computed to ensure consistency of the interactions against random effects and thus point out robust mRNA–ncRNA associations. Significant bio-annotations (edge label color) were also reported to specify the functional contexts, those putatively linking the ncRNAs to functions through their interacting genes (see Supplementary Materials Files S4 and S5).

### 3.3. Functionalization of the Maps

The most relevant nodes emerging from the network maps are the high-brokerage ncRNAs (see Supplementary Materials File S6). In the basal map (Figure 2), DBF4P, ADH5P3, PCNAP3, UBE2SP1 are highlighted, while in the luminal map (Figure 3), the selected ncRNAs are: TNXA, AQP7P4, FGF14AS2, GAPDHP65, RRM2P4. These detections present sufficient annotation in current catalogs and repositories from either literature or in relation to experiments. Many other ncRNAs were profiled quantitatively but without annotation (not yet cataloged or unknown). The most prevalent ncRNA biotype is represented by pseudogenes, which is expected as pseudogenes have regulatory power that has been established in the literature to a larger extent compared to other ncRNAs.

### 3.4. Subtype-Specific Biological Contexts

At a first glance, both the basal and the luminal maps (Figure 2 and Figure 3) show a blend of biological influences from a variety of contexts associated with the highlighted ncRNA. A summary of them is provided next.

#### 3.4.1. Basal Cell Cycle Context

The two main ncRNA players involved are ADH5P3 and especially PCNAP3. Apart from referring to cell cycle promoters [36], the functional relevance of the ADH5P3 appears through its interactions: (a) UBE2C (*Ubiquitin conjugating enzyme E2 C*), with ubiquitination involving various enzymes in part involved with mitotic cyclins and cell cycle progression (b) MMRN2 (*Multimerin 2*), negative regulator of angiogenesis (c) CORO2A (*Coronin 2A*), relevant for cell cycle progression, apoptosis, signal transduction, gene regulation (d) FOXM1 (*Forkhead Box M1*), whose encoded protein is a transcriptional activator involved in cell proliferation (e) CDCA3 and 5 (*Cell Division Cycle Associated 3 and 5*) (f) MKI67 (*Marker of Proliferation Ki-67*). In the PCNAP3 contextual interactions are with: (a) CDCA3 and CDCA5, (b) UBE2C, (c) FAM83D (*Family with sequence similarity 83 member D*), whose regulation involves proliferation and growth together with migration and epithelial to mesenchymal transition (d) CDC20 (e) CCNA2 (*Cyclin A2*), main regulator of cell cycle, and (f) MELK, a kinase involved in various processes including cell cycle regulation.

Considering the other two ncRNA players, DBF4P1, and UBE2SP1, the cell cycle context involves other interactors such as UBE2C, FAM83D and MKI67, together with BUB1B (*Mitotic Checkpoint Serine/Threonine Kinase B*), which encodes a kinase involved in spindle checkpoint function whose impairment is found across cancers, negatively regulating the interactor PLK1 (*Polo Like Kinase 1*). PLK1 depletion in cancer cells inhibits cell proliferation and induces apoptosis; thus, this is a target for cancer therapy.

#### 3.4.2. Basal Chromatin Context

This specific context emerges from the annotated interactions for ADH5P3 and PCNAP3. The first, ADH5P3, present these relevant interactors: (a) CDCA5, related to chromatin binding; (b) CBX7 (*Chromobox 7*), containing the chromatin organization modifier domain; (c) LMNB1 (*Lamin B1*); (d) TPX2, a microtubule nucleation factor required for chromatin-dependent microtubule nucleation. For PCNAP3, apart from the links with TPX2, LMNB1, and CBX7, it is worth mentioning ASF1B (*Anti-silencing Function 1B Histone Chaperone*), which plays a role in modulating the nucleosome structure of chromatin by supplying histones. For DBF4P1 and UBE2SP1, the cell cycle is again enriched via LMNB1.

#### 3.4.3. Other Basal Functional Contexts

The DNA damage and cytoskeletal signaling context are relevant for ADH5P3, via CEPP55 (*Centrosomal Protein 55*), and for DBF4P1, via CENPF (*Centromere Protein F*) and KIF20A (*Kinesin Family Member 20A*). The immunoregulation context is present through ADH5P3 interacting with CXCL14 (*C-X-X Motif Chemokine Ligand 14*) and through JAM2 (*Junctional Adhesion Molecule 2*). The metabolism context appears with PCNAP3 interacting with FAM72A *(Family with sequence similarity 72 member A*) and CALCOCO1 (*Calcium Binding and Coiled-Coil Domain 1*). Finally, the apoptosis context is relevant for PCNAP3 via the interaction with BIRC5 (*Baculoviral IAP Repeat Containing 5*), a member of the inhibitor of apoptosis (IAP) gene family and negative regulator preventing apoptotic cell death.

Looking at the whole map, UBE2SP1 appears to be the most influential node of the basal sub-network, as it spans some of the previously mentioned contexts, especially metabolism through IQGAP3 (*IQ Motif Containing GTPase Activating Protein 3*), ubiquitously overexpressed in several human cancers, and ARHGAP11A (*Rho GTPase Activating Protein 11A*), relevant for cell cycle arrest and apoptosis and specifically known to be highly expressed in a human basal-like BC cell line. The influences reach DNA damage and cytoskeletal signaling, but also chromatin-related interactions such as ASF1B, AURKA, and AURKB (*Aurora Kinases*) and CDCA5, then CDK1 (*Cyclin Dependent Kinase 1*), which has an important cell cycle control role also associated with BC and finally SPARCL1 (*SPARC Like 1*), relevant for transport via calcium ion binding.

### 3.5. Major Luminal Contexts (Transport, Angiogenesis, Cell Migration, Cell Adhesion)

In the luminal sub-network, five ncRNAs are highlighted: TNXA, AQP7P4, FGF14AS2, GAPDHP65, RRM2P4. Of particular interest is the integrated functional context involving three players. FGF14AS2 (*FGF14 Antisense RNA 2*) is affiliated with the lncRNA class and BC is among its associated diseases. Functions annotated as transporter and potassium channel activities emerge via AQP1 (*Aquaporin 1*), a water channel membrane protein that promotes tumor angiogenesis (new blood vessel formation) by allowing faster endothelial cell migration. This bridges with the more centrally located pseudogene TNXA.

Then, an interesting triangulation appears between FGF14AS2 with AQP1 and MYL9 (*Myosin Light Chain 9*), involved in calcium ion binding and for which the subnetwork is reported at the lower right, bridging with AQP7P4. Here, we again find the angiogenesis functional context involved through GPR124 (*G protein-coupled receptor 124*), also identified as a ligand-specific coactivator of canonical Wnt signaling, APCDD1, a negative regulator of the Wnt/β-catenin signaling involved in angiogenesis and barrier formation, and finally ADCY5. This adenylate cyclase (AC) increases the intracellular levels of cyclic AMP (cAMP), indicating that a major signaling pathway that facilitates angiogenesis is cAMP-dependent. Angiogenesis is also enriched by AMOTL2 (*Angiomotin Like 2*), which binds angiostatin, a circulating inhibitor of angiogenesis, and by SRPX, which induces endothelial cell migration, adhesion, and the formation of a vascular network. Finally, ADAM33 (*ADAM Metallopeptidase Domain 33*) is relevant in terms of cell migration, cell adhesion, cell–cell, and cell–matrix interactions, and signal transduction.

Regarding the whole map, TNXA induces a hub that enriches a variety of biological processes relevant to cancer. For instance, cell adhesion and angiogenesis via (a) BOC (BOC *Cell Adhesion Associated Oncogene Regulated*), (b) ID4 (*Inhibitor of DNA Binding 4*, *HLH Protein*), implicated in regulating a variety of cellular processes including cellular growth, senescence, differentiation, apoptosis, angiogenesis, and neoplastic transformation, and (c) SRPX and GPR124 (see above). Then, a group of growth factors is enriched, such as MRGPRF (control of cell proliferation), PDGFRL (platelets), and HIC1 (growth regulatory and tumor repressor gene). Of special interest is SERPING1, which plays a potentially crucial role in regulating important physiological pathways, including complement activation, and has been studied to understand the context-dependent role of complement and immune-related transcriptome profiles of primary tumors and lymph node metastases, especially in luminal BC [37]. Other interesting interactors refer to SOX7, which has a role in tumorigenesis and is part of transcription factors family involved in determining the cell fate, and CBX7, earlier described in the basal sub-network and here involved with the PcG PRC1 complex in chromatin remodeling and modification of histones.

#### 3.5.1. Luminal Cell Cycle Context

RRM2P4 appears as a major hub located at the top of the map, which involves a context characterized by cell cycle and centered on UHRF1 (*Ubiquitin Like with PHD and Ring Finger Domains 1*). Then SPAG5 (*Sperm-associated antigen 5*) is present, a novel oncogene in various cancers and highly expressed in BC, although its biological function and regulatory mechanism are unclear. In a recent study, SPAG5 was shown to promote both proliferation and invasion of BC cells by activating Wnt/β-catenin signaling via the upregulation of Wnt3 expression [38]. Apart from the cell cycle context, it is particularly relevant in the interaction with RAD51 (*RAD51 Recombinase*), which shares activity with BRCA1 and BRCA2 affecting the cellular response to DNA damage. For example, BRCA2 regulates its intracellular localization and DNA-binding ability and the loss of control following BRCA2 inactivation may lead to genomic instability and tumorigenesis.

#### 3.5.2. Luminal Metabolism Context

The ncRNA GAPDHP65 (*Glyceraldehyde 3 Phosphate Dehydrogenase Pseudogene 65*) is related to the already known GAPDH regulation of glycolysis and has a pivotal role in tumor metabolism [39], which also explains why GAPDH represents an attractive target for therapeutic intervention. For instance, it plays a crucial role in the maintenance of cellular redox balance [40], in protecting the cells from free radical or ROS-mediated injury, and in both pro-apoptotic and oncogenic processes. However, the identification of the intracellular mechanism directing its proapoptotic or oncogenic role remains unclear [41]. This pseudogene interacts with other functional contexts (angiogenesis, cell migration, transport) that have been highlighted with the subnetworks of the already described hubs AQP1 and MYL9, and also interacts with PBK, which has a protein kinase related to the dual-specific mitogen-activated protein kinase (MAPKK) family.

### 3.6. Validations

#### 3.6.1. RNA/Protein Correlation

In general, subtype-specific networks show differentiated core signaling mechanisms [42]. The maps previously introduced integrate additional genomic complexity through the potential ncRNA-induced regulations over contextual genes and their annotated processes/pathways. Although computationally validated, the ncRNA brokers may serve as putative prognostic biomarkers and therapeutic targets until further validations are produced.

**Figure 4:**
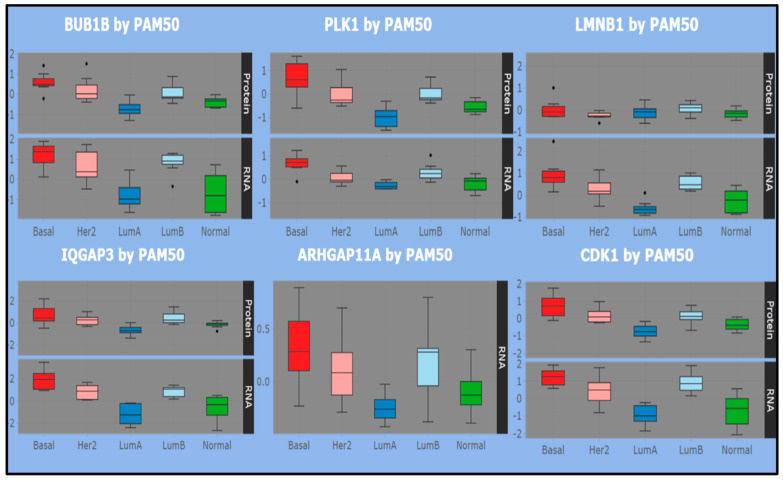

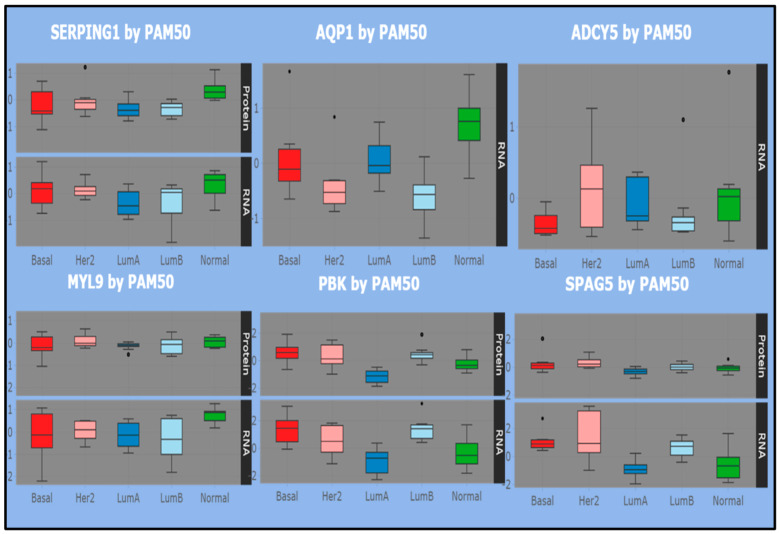
RNA–Protein correlations for some selected target genes/proteins (basal map top panel, luminal panel bottom panel). Boxplots of significant results. Average expression values reported in samples grouped by PAM50 subtypes. Source of the plots: https://www.breastcancerlandscape.org/, accessed on 23 November 2021.

Recently, a proteogenomic landscape was built and online resources have become available for validating evidence and cross-referencing studies [43]. From their use, positive and significant correlations were found across tumors between 70% of the proteins quantified in the *Oslo2 Landscape* cohort and their mRNA transcripts. Therefore, it is reasonable to use this proteogenomic information to validate the identified ncRNA contextual genes subject to brokers’ influence in the basal and luminal maps. Some significant correlative examples are reported (Table 2), while Figure 4 displays significant statistics (boxplots) for some key associations established across the reference subtypes.

Overall, highly proliferative tumors (basal-like and luminal B) tend to show more correlated proteomes and transcriptomes than lowly proliferative tumors (luminal A). Specifically, cell cycle is associated with high tumor mRNA–protein correlation. This is clearly seen in the top panel of Figure 4, with reference to the basal map (top panel), where RNA–protein correlations are established between positive average expression levels of selected genes/proteins. Instead, LMNB1 uniquely shows correlation of negative expression value for the same subtype (considering that ARHGAP11A has only RNA measurement). The same genes are also measured in other subtypes, including luminal A/B. Correlated RNA–protein negative expressions appear only for luminal A with reference to BUB1B, PLK1, IQGAP3, CDK1, while luminal B shows correlations more aligned with the basal subtype and discordant with luminal A. Then, although LMNB1 is not easy to discriminate, the role of these genes is well justified in the ncRNA-driven basal map with a cell proliferation signature and strong cell cycle biological context, while such evidence is lacking in the luminal map.

In the luminal map (bottom panel), the picture revealed by the distribution of the selected genes/proteins across the listed subtypes is generally more heterogeneous. SERPING1 and MYL9 fluctuate between positive and negative expression levels in the basal subtype, while PBK and SPAG5 present more positive expressions. All such entities are correlated in RNA and protein expressions, but with negative average levels in luminal A, while luminal B is concordant with that observed in the basal subtype for SERPING1 and MYL9 and with the more positive expression levels for PBK and SPAG5.

Overall, the RNA/Protein correlation holds across subtypes, with signs changing between basal and luminal A/B, the latter exhibiting a mix of concordant and discordant boxplot pattern. In conclusion, highlighting the RNA–Protein correlation component may reveal useful information for elucidating the role of some genes in obtaining a network submodular characterization.

#### 3.6.2. Prognostic Impact (Survival)

With the UALCAN [44] tool it is possible to explore the relationships between sub-group gene expression and survival analysis. We used it to validate hub genes as prognostic biomarkers and therapeutic targets for BC, although other validations are also possible (for instance, by proteome profiling [45]).

**Figure 5:**
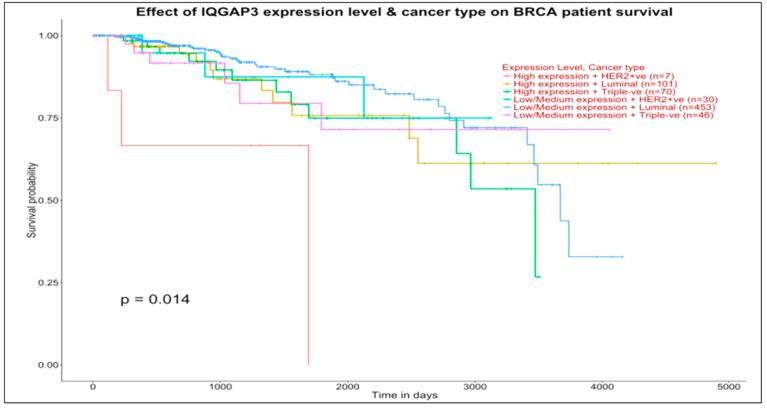

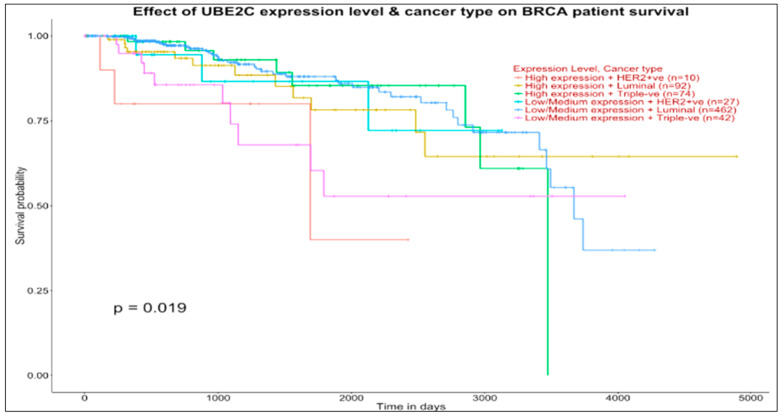
Basal selections with prognostic impact on breast invasive carcinoma (BRCA) patients. Source: UALCAN. Significance of survival impact is measured by log rank test (the lower the *p*-value, the better the separation between groups).

**Figure 6:**
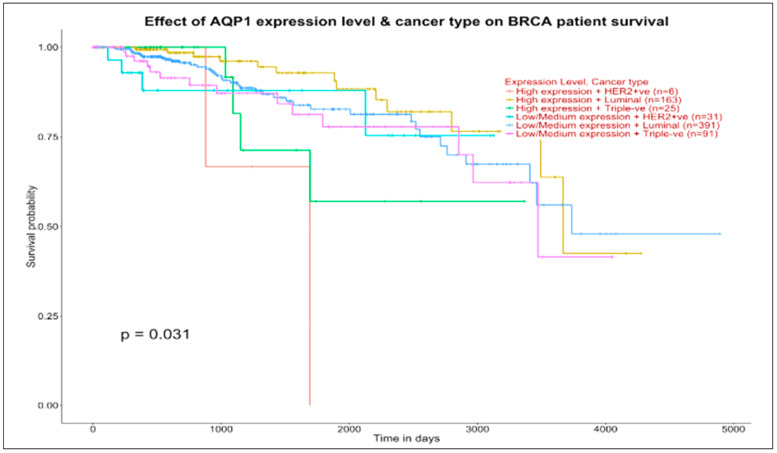

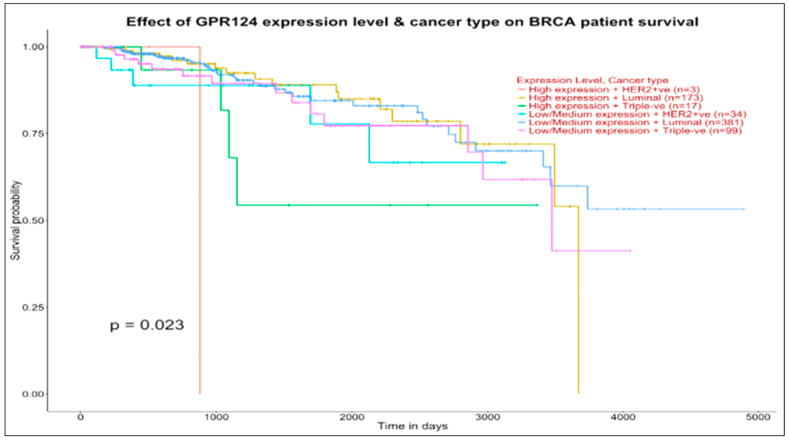
Luminal selections with prognostic impact on breast invasive carcinoma (BRCA) patients. Source: UALCAN. Significance of survival impact is measured by log rank test (the lower is the *p*-value, the better is the separation between groups).

Figure 5 and Figure 6 show the effects (measured with *p*-values from the log rank test informing the subtype group separation, i.e., the lower is the *p*-value, the better is the separation between groups) on patient survival of single genes previously examined in our two network maps. This analysis is therefore targeted. The top panel of Figure 5 (IQGAP3, target of UBE2SP1 in the basal map) shows overall differentiated survival patterns among various subtypes, with approximately concordant curves between:

- Luminal low-medium expression and triple-negative high-expression groups (453 vs. 70 patients, respectively), with survival after 4000 days for the first group and marginally after 3500 days for the second group)
- Luminal high expression and triple-negative low-medium expression groups (101 vs. 46 patients, respectively), with quite long survival time measured in both groups.

Similar curve concordance between these groups is observed at the bottom panel (UBE2C, which interacts with PCANAP3, ADH5P3, and DBF4P1 in the basal map) with 462 vs. 74 patients (first comparison) and 92 vs. 42 patients (second comparison).

When we look at the top panel of Figure 6 (AQP1, target of AQP7P4, GAPDHP65, FGF14A52, and TNXA in the luminal map), we observe survival pattern differentiation and even more generalized curve concordance between the four examined groups, all with survival probability just around 0.5 beyond 3000 days.

Things change when we consider the bottom panel (GPR124, target of both TNXA and AQP7P4 in the luminal map), with curve concordance at low-medium expression levels in luminal and basal groups and with the high expression groups indicating a survival ending close to 4000 days for luminal (173 patients) and a survival above 0.5 approaching 4000 days for basal (17 patients). In conclusion, the survival curves examined through the effects of targeted genes contextual to the ncRNAs and previously investigated in the maps may be prognostically informative.

#### 3.6.3. Regulation of Transcription Factors

**Figure 7:**
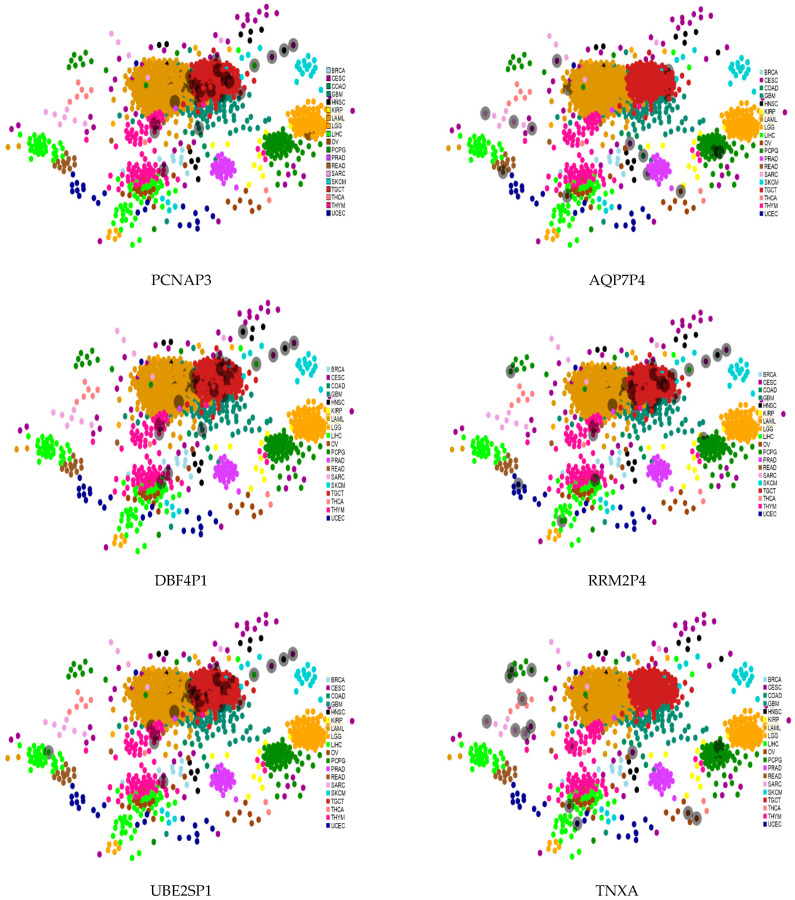
Examples of TF maps centered on basal (**left** side) and luminal (**right** side) selections. Source: ChEA3. Top 50 TF selected with integrated rank across libraries (ChIP-Seq with ENCODE, ReMap and Literature, co-occurrence with Enrichr, co-expression with GTEx and ARCHS4).

Transcription factors (TFs) enable pervasive regulation and in Figure 7 we mapped TFs referring to some of the ncRNA-driven biological contexts across TCGA cancers. The TFs populating the maps are retrieved from the ncRNA direct interactors using the ChEA3 (ChIP-X Enrichment Analysis 3) tool with its large collection of gene set libraries generated by multiple sources that are then integrated to yield better TF prediction via a composite rank [46]. The cancer coverage by TF (grey shadowed dots in the maps) appears more heterogeneous for the luminal map (right side figures). Partial similarity is also noted across TF in both subtype maps; especially, RRM2P4 is more aligned than both AQP7P4 and TNXA with all the basal (left side figures).

## 4. Discussion

Unravelling the molecular mechanisms potentially linked to cancer is complicated due to the heterogeneity of the processes underlying this disease. Despite several studies targeting common influences, the definition of a landscape of global regulatory pathways affecting multiple cancer types or even subtypes remains hypothetical, even when considering the same cancer [10]. The heterogeneity partially captured by identified molecular subtypes usually responds to applied unsupervised clustering of gene expression profiles then correlated with phenotypic, histological, and clinical variables.

Recently, other strategies have integrated multiomics data [47] and suggested that, in order to contrast the data heterogeneity bottleneck, subtype separation should occur in a data-integrated space informing on copy number variation, mRNA expression, and protein levels. However, a space accounting for ncRNA remains problematic. Despite the functional potential of ncRNA biotypes, predicting their functions without well-annotated features is beyond reach. Our approach is an attempt to use network topology in support of ncRNA functional prediction efforts. There are inherent limitations in our approach. Our node attributes referring to a wide-spectrum transcriptome coverage yielding gene expression profiles that can just shape baseline topological configurations and govern the information flow represented by networks at only limited interaction level, as the maps that we produced could have been enriched by genetic interactions and microRNA interactions (with gene targets).

Additionally, the ncRNAs that we map onto the networks may bind to single proteins or complexes in quite different ways and thus regulate functions at different complexity levels. This indicates that the ncRNAs may have multiple roles in tumorigenesis and their induced interaction dynamics can be context-dependent and cell-dependent [5,12]. It is certain that the ncRNA functions can be corrupted by the presence of cancer in multiple ways (fusion of oncogenes etc.) [3]. In general, the evidence here obtained may benefit from a key factor, i.e., scale. Further validation experiments to verify functional hypothesis and clinical applicability in the BC domain may need additional dataset sources and the generalization of the proposed approach to other cancer types may be an interesting direction for research. Ultimately, the network maps can represent templates centered on ncRNAs from which functional hypotheses can be generated in silico and then used to guide further testing and validation.

Finally, a generally debated issue is that the 50-gene signature, PAM50, was originally defined to identify subtypes in clinical setting by using unsupervised statistical methods and leaving aside aspects such as the biological relevance of the selected genes, including their regulatory drivers in context with many regulation sources. In light of the importance acquired by ncRNAs within the omics data dimensions, it is clear that another weakness concerns how to associate identified ncRNAs to the subtypes [19]. Assuming that some sort of accurate inference can obtained, the question then becomes what validation to use. The identification of the functional role of ncRNAs would require direct perturbation experiments. Despite the difficulties of such experiments, some functional characterizations have been obtained. In principle, in silico computational analyses may represent valid inference support tools guiding perturbative experimental settings [14].

## 5. Conclusions

The simple idea presented in this work is that the methodology here described can indicate how gene signatures can be more robust. Biclustering is a well-known approach combining genes and sample into data partitions that facilitate the identification of markers given sufficient sample size and significance of the gene dysregulation effects that are captured. We proposed the use of the biclusters as usable templates for determining regulatory modules, knowing that the latter are subject to multiple sources of influence. Nevertheless, the network modules that we obtained from these templates offer the potential to increase the robustness by transforming the weak signals associated with one-step clustering-driven gene signatures correlated with outcome [48]. For subtyping BC samples, our approach leverages the putative role of ncRNAs in regulating the network dynamics inherent to selected biclusters, i.e., those with subtype dominant signature.

Pseudogenes, a rich family of ncRNAs associated with mRNA targets, appear as the most significant biotype that our methodology prioritizes at the topological level through brokerage. Brokerage is one of the methods that have been investigated to detect network vulnerability. Articulation points is another method for considering nodes for which removal disconnects the network [29]. In general, a bridge is a cut-edge, or an edge in a graph whose removal disconnects it, while an articulation point is a cut-vertex, or a node in a graph for which removal disconnects it. Notably, they both represent vulnerabilities of networked systems. As a reminder, to quantify the importance of an edge in damaging a network, bridgeness is useful to account for the number of nodes disconnected from the giant connected component after the removal of the edge [30].

However, a general framework for studying such types of nodes and the network vulnerability associated with them is currently lacking. Brokerage exerts a form of control over bridging and can reduce the overall network cohesiveness, especially with nodes/links of high betweenness and/or able to disconnect a graph when removed. In this work, we have determined that ncRNAs can disrupt BC subtype networks and have established that by checking the property of bridgeability (the extent to which the network is characterized by one or few brokers/bridges [49]) in such cancer networks, we can measure structural effects that generally influence function and, more specifically, phenotypes. Consequently, the more brokers (ncRNA and non) that populate the cancer subtype networks, the more these become vulnerable to attacks directed to them, thus becoming dependent on relatively few regulating structures to remain functional. Measuring the stability of such regulators under different types of perturbations would provide useful diagnostic information. In conclusion, we stress the potential generalizability of our findings to various cancer types, as well as the translational relevance of our approach based on the accurate and robust identification of cancer targets that can refine possible validation strategies.

## Figures and Tables

**Table 1:** ISA biclusters with PAM50 molecular subtypes. Top biclusters highlighted in each subtype.

Bicluster	Luminal A	Luminal B	Normal-like	Basal
#1	0.10	0	0.17	**0.73**
#2	0.12	0.29	0.46	0.12
#3	0.27	0.12	0.38	0.23
#4	0.07	0.36	0.32	0.25
#5	0.21	0.25	0.42	0.12
#6	0.04	**0.46**	0.35	0.15
#7	0.04	0.40	0.40	0.16
#8	0.04	0.42	0.42	0.12
#9	0.04	0.42	0.38	0.16
#10	0.08	0.24	0.48	0.20
#11	0.03	0.37	0.29	0.31
#12	**0.29**	0.14	0.43	0.14
#13	0.05	0.33	**0.57**	0.05

**Table 2:** Established RNA–Protein correlations (source https://www.breastcancerlandscape.org/, (accessed on 23 November 2021).

Gene Symbol	mRNA–Protein Correlation (Spearman)	*p*-Value	Adj *p*-Value (Bonferroni)
SPAG5	0.5804	4.10 × 10^−5^	0.000144479
SERPING1	0.3311	0.026795892	0.039512315
MYL9	0.5045	0.000486773	0.00119107
LMNB1	0.4149	0.004911333	0.008801109

## Data Availability

All data appear in the Supplementary Files. For reproducibility scope, methods are available from the authors.

## Supplementary Materials

The following supporting information can be downloaded at: https://www.mdpi.com/article/10.3390/jcm11082103/s1, SM File S1, data; SM File S2, stability analysis; SM File S3, Venn Diagrams; SM File S4, Basal GO processes; SM File S5, Luminal GO processes; SM File S6, ncRNAs.
