# Supplementary material for: Non-Coding RNAs Are Brokers in Breast Cancer Interactome Networks and Add Discrimination Power between Subtypes
Source: J Clin Med. 2022 Apr 9;11(8):2103. doi: 10.3390/jcm11082103 (PMC9029160; doi:10.3390/jcm11082103)
Supplement: Supplementary file 1 [file jcm-11-02103-s001.zip › SM File S2 Stability.pptx]

## Slide 1
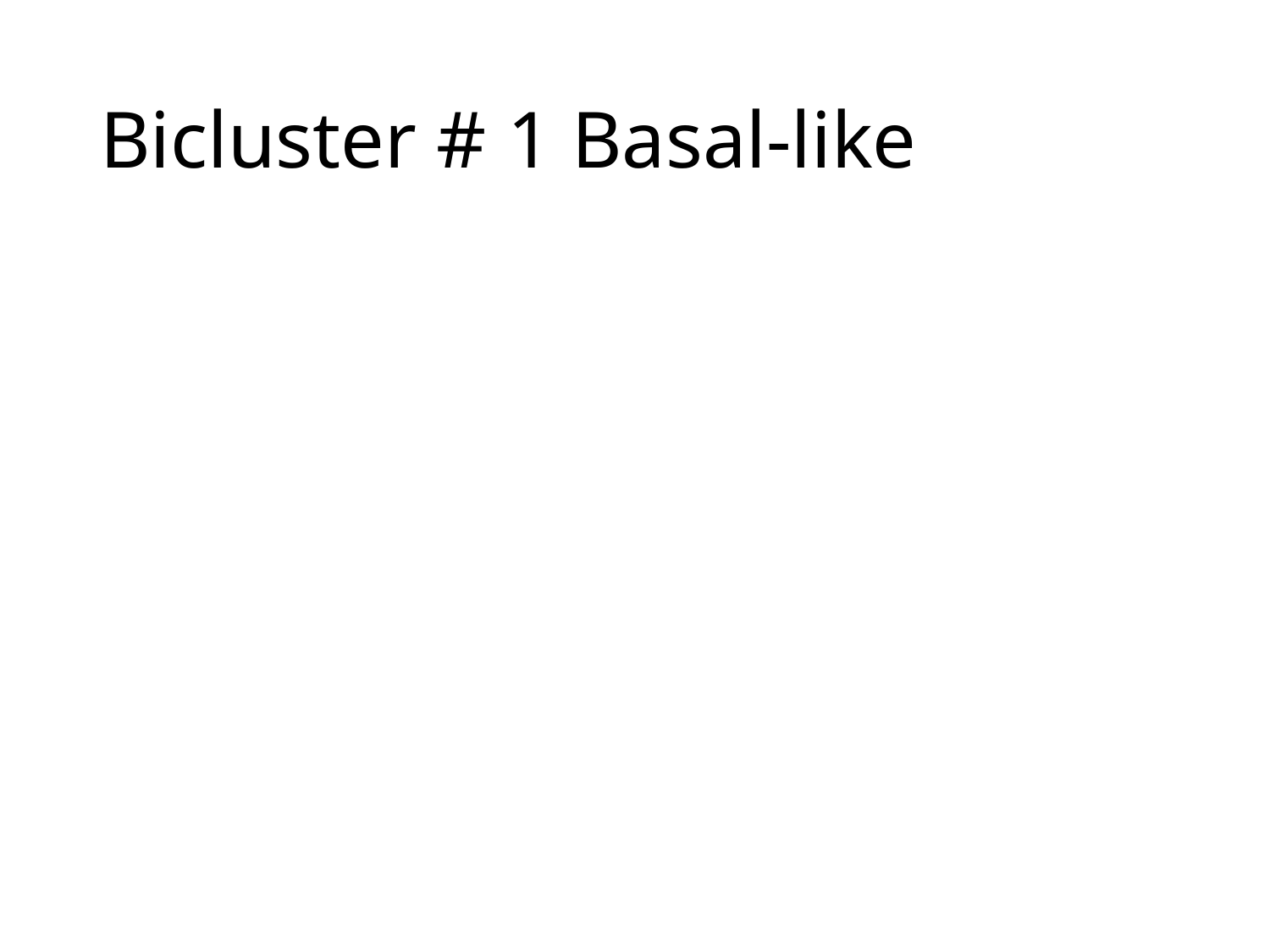

# Bicluster # 1 Basal-like

## Slide 2
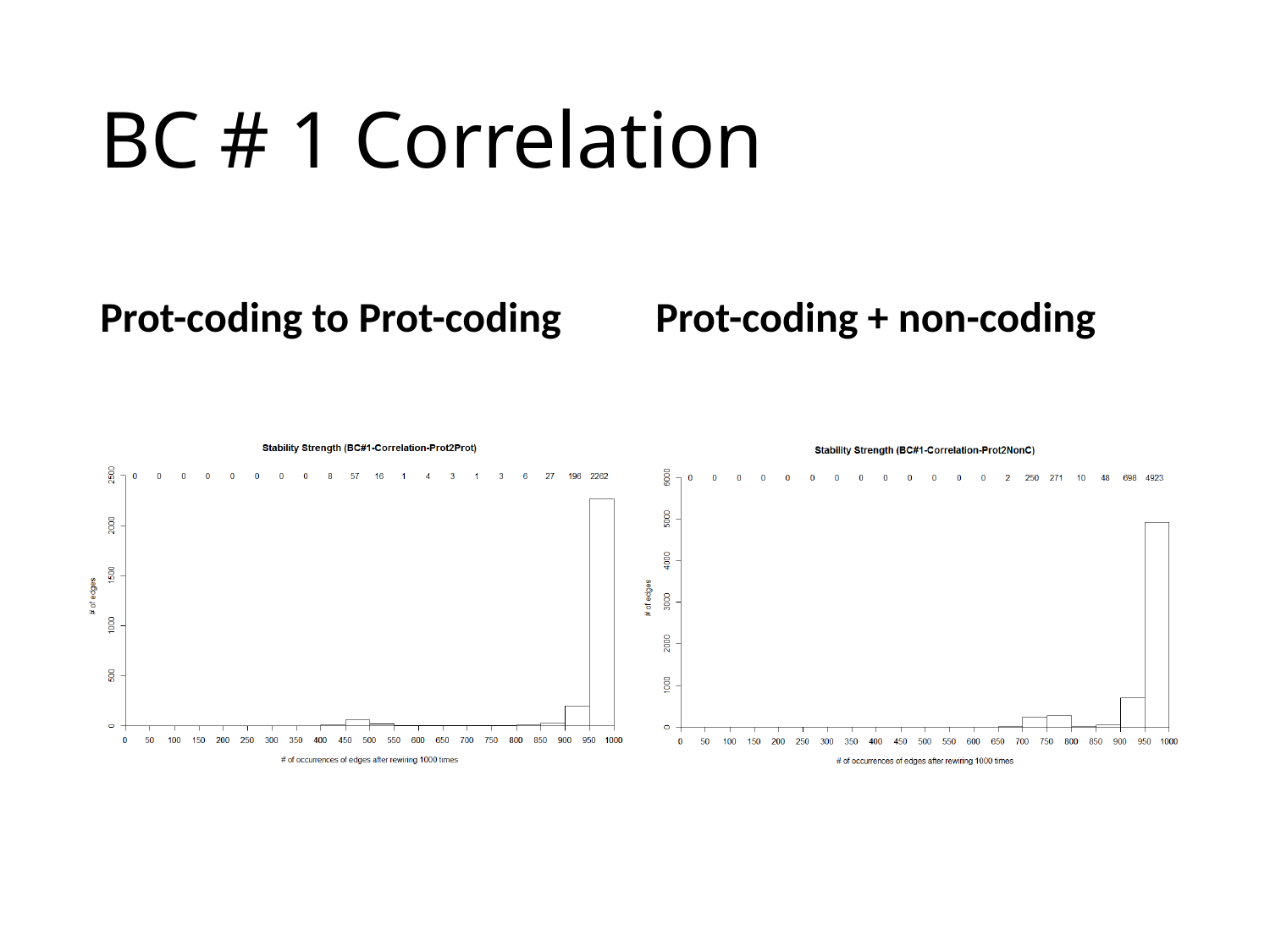

# BC # 1 Correlation
Prot-coding to Prot-coding
Prot-coding + non-coding

## Slide 3
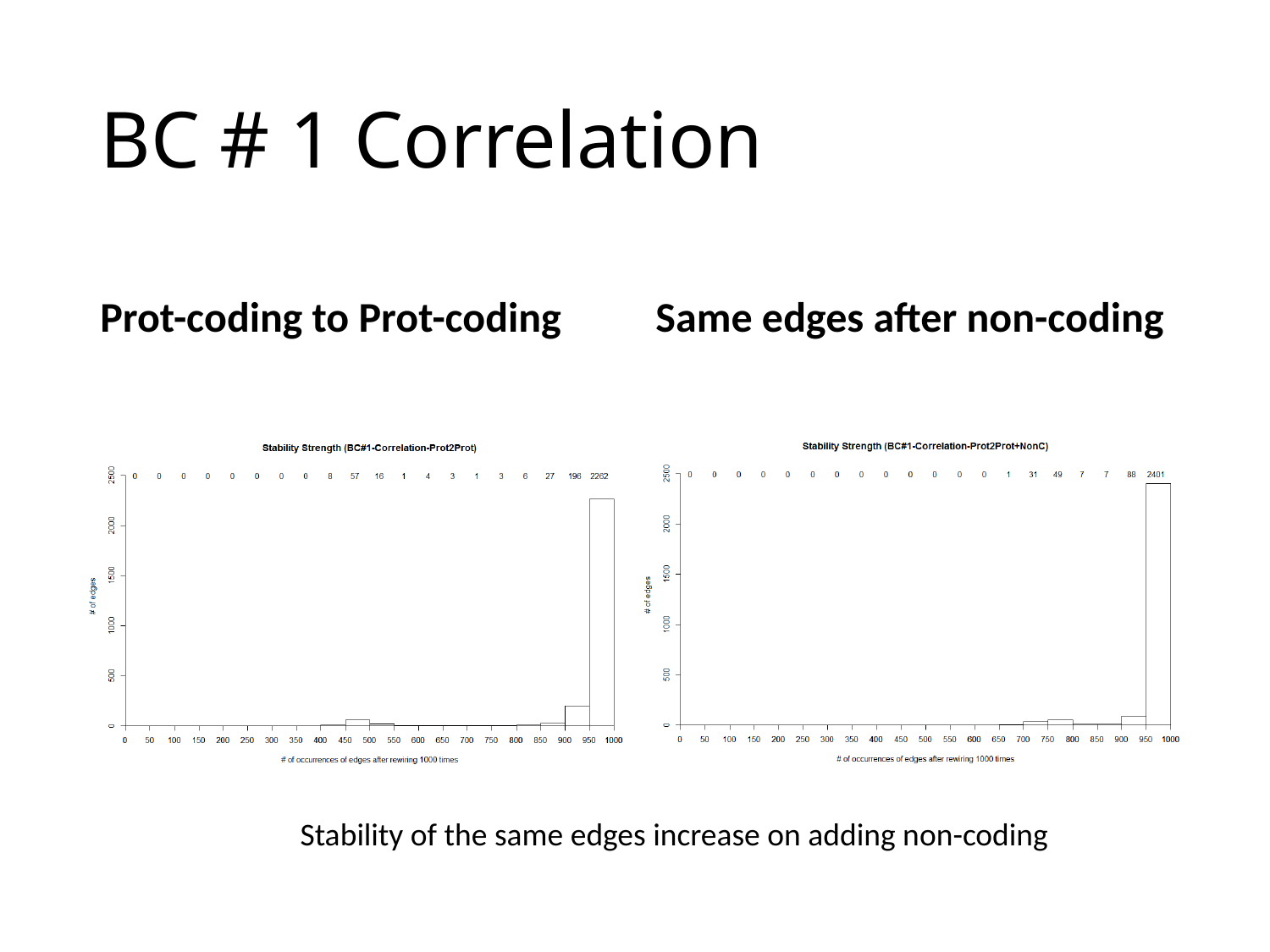

# BC # 1 Correlation
Prot-coding to Prot-coding
Same edges after non-coding
Stability of the same edges increase on adding non-coding

## Slide 4
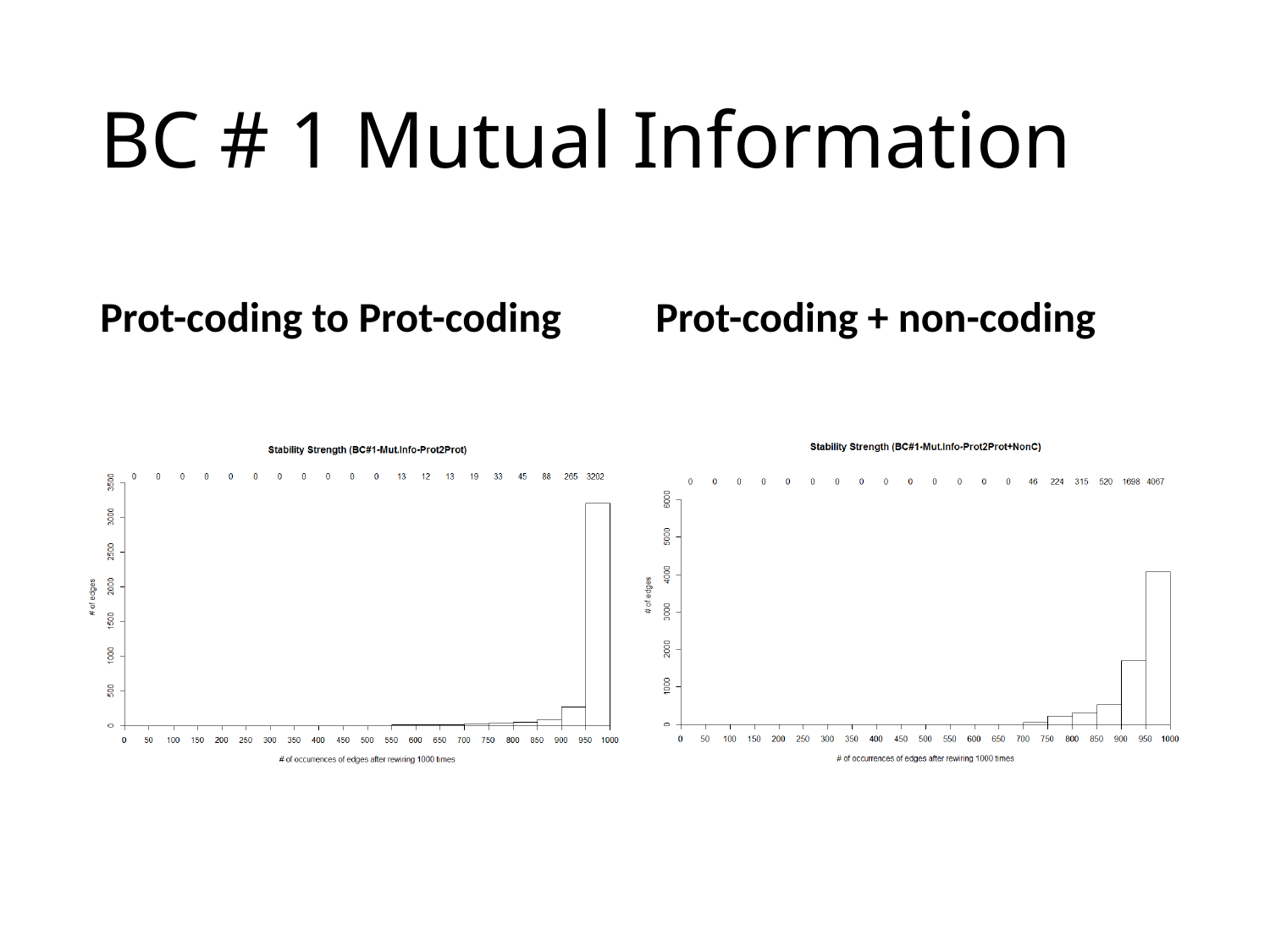

# BC # 1 Mutual Information
Prot-coding to Prot-coding
Prot-coding + non-coding

## Slide 5
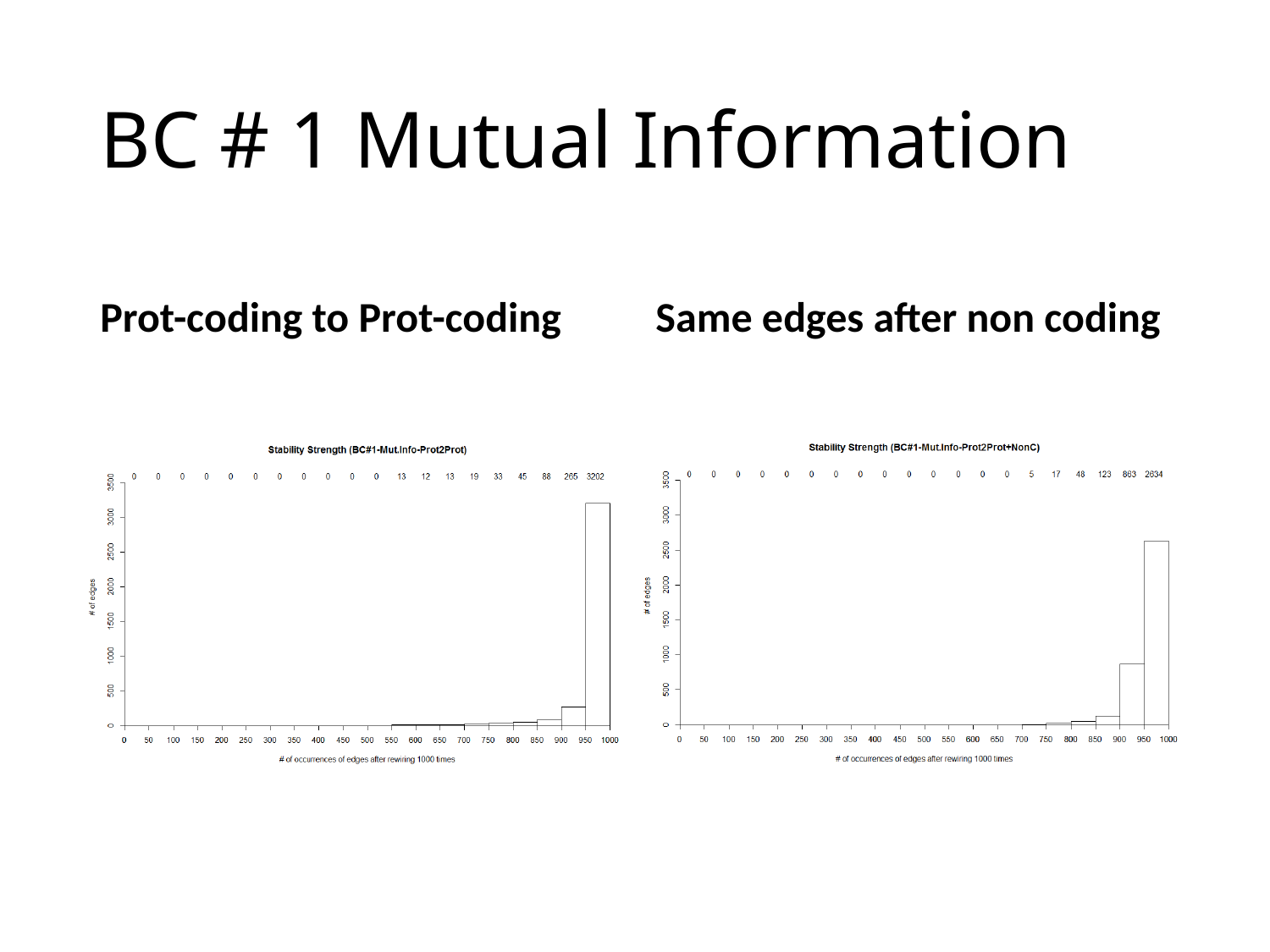

# BC # 1 Mutual Information
Prot-coding to Prot-coding
Same edges after non coding

## Slide 6
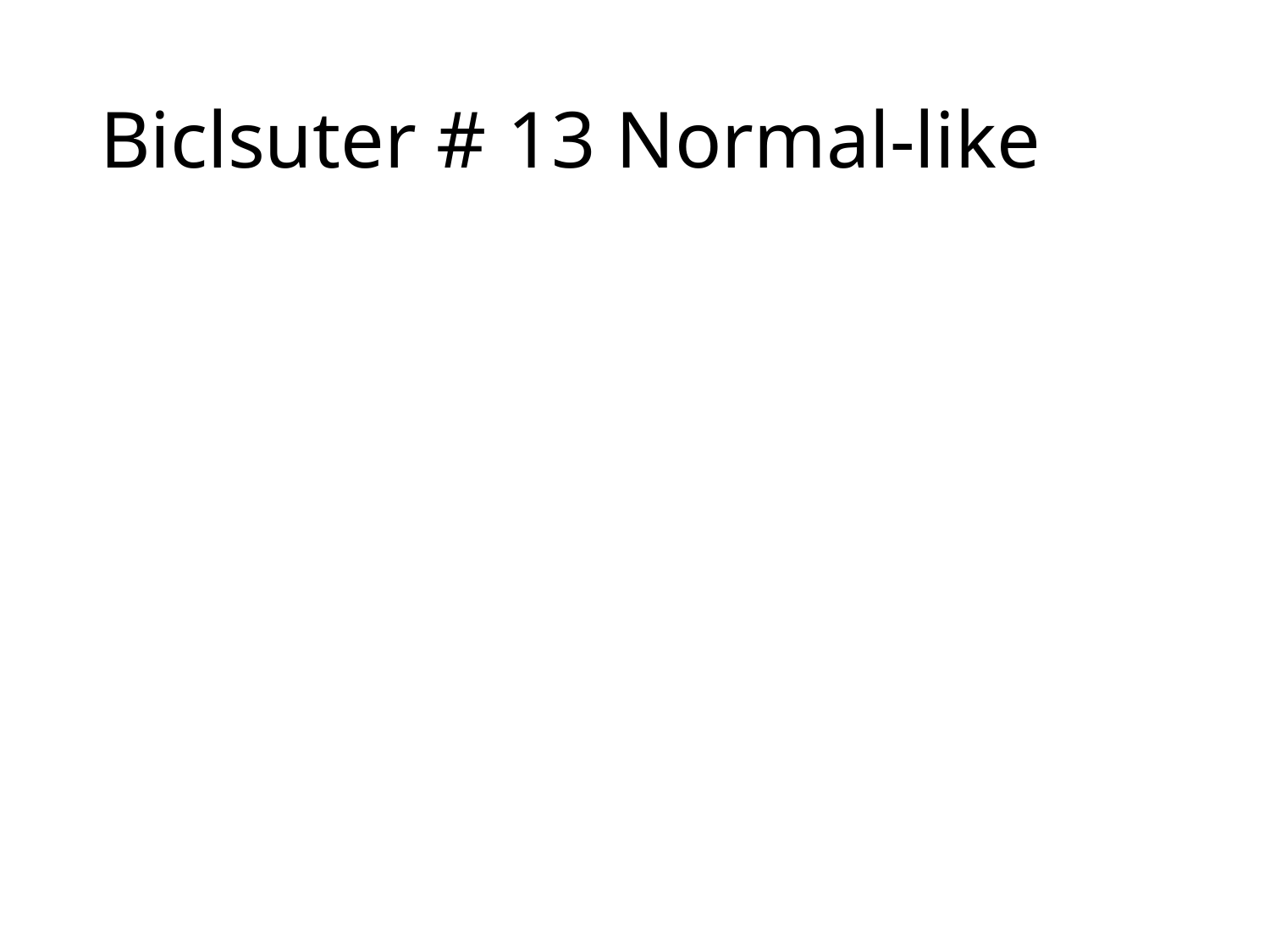

# Biclsuter # 13 Normal-like

## Slide 7
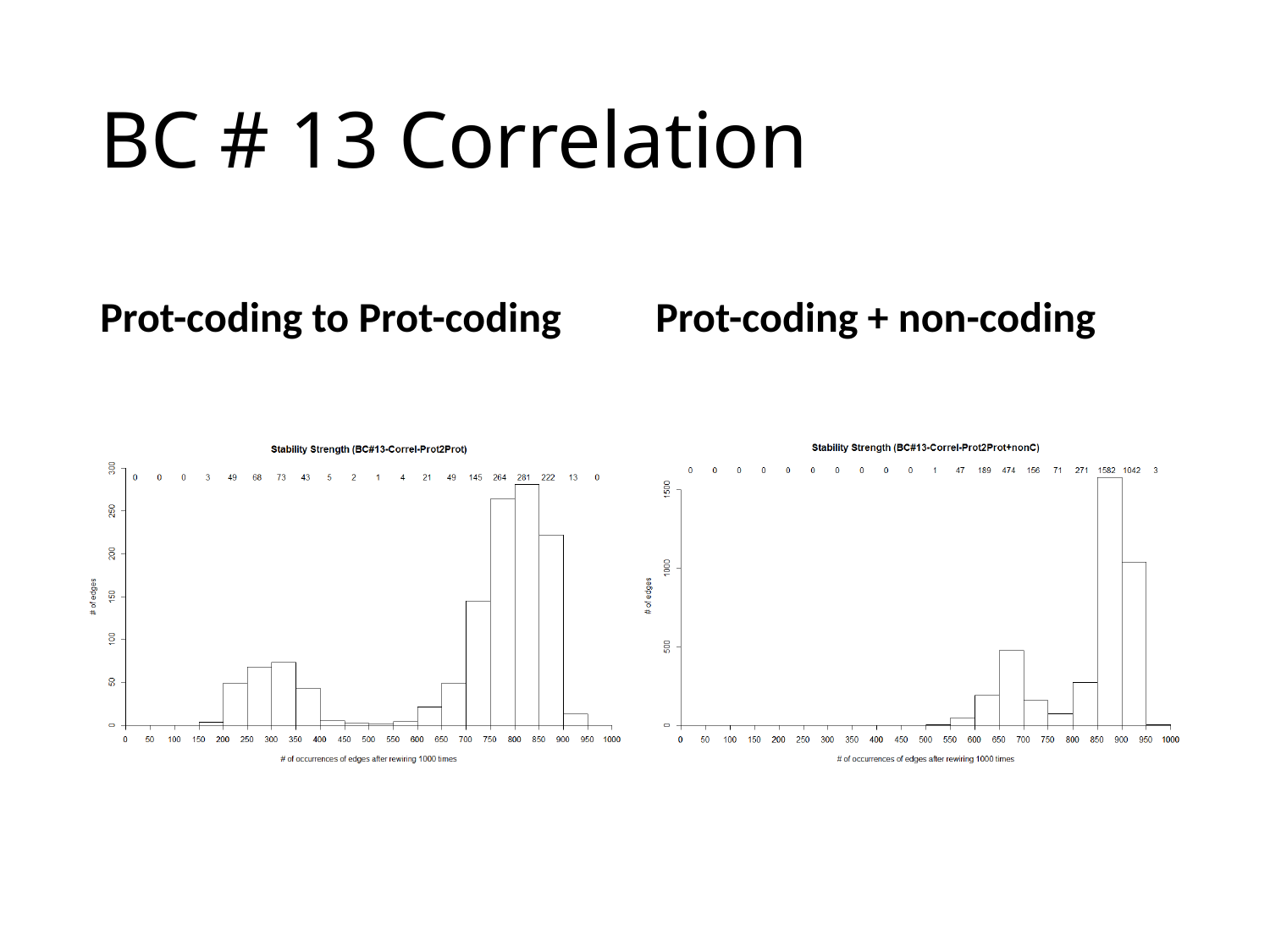

# BC # 13 Correlation
Prot-coding to Prot-coding
Prot-coding + non-coding

## Slide 8
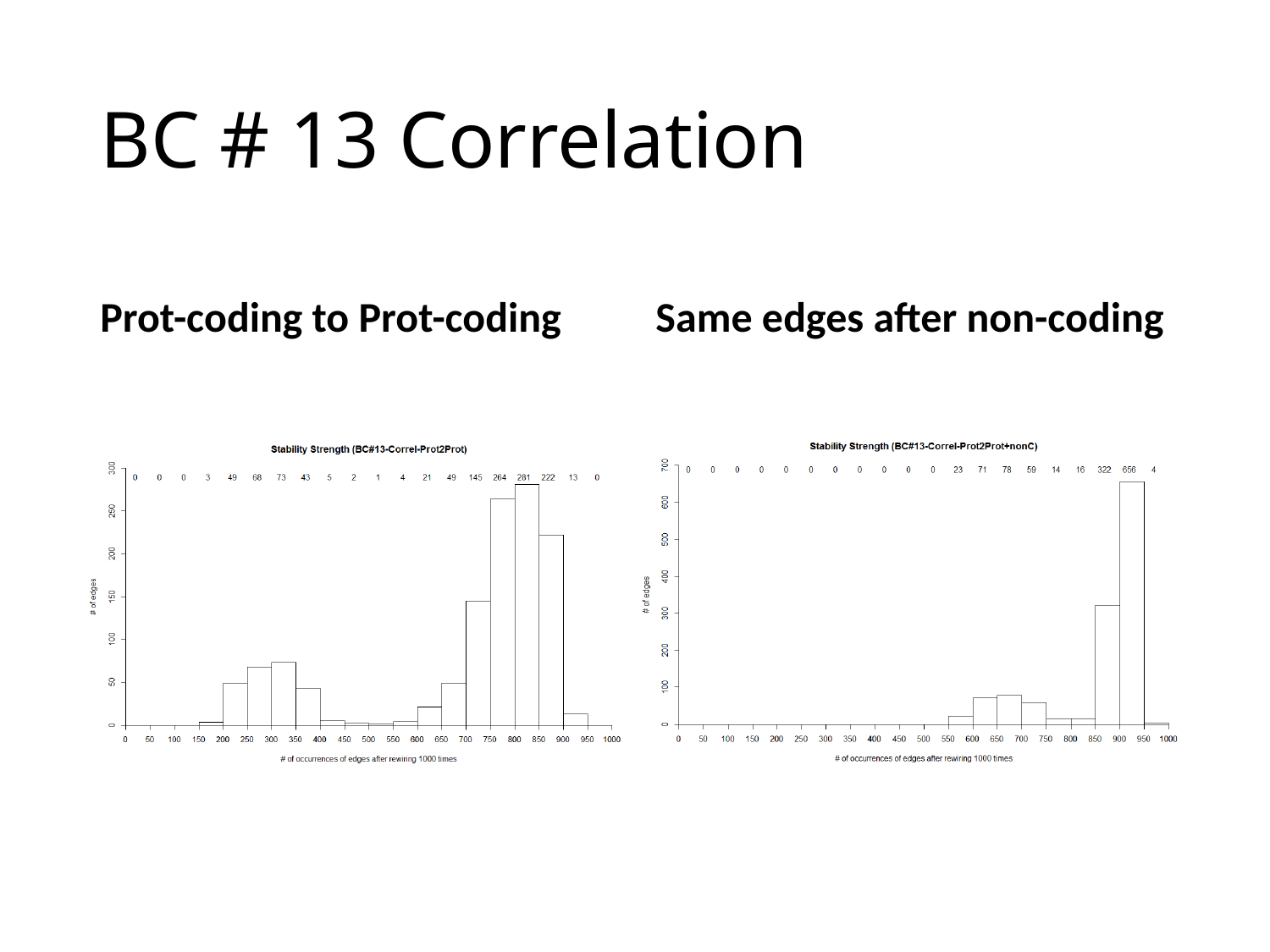

# BC # 13 Correlation
Prot-coding to Prot-coding
Same edges after non-coding

## Slide 9
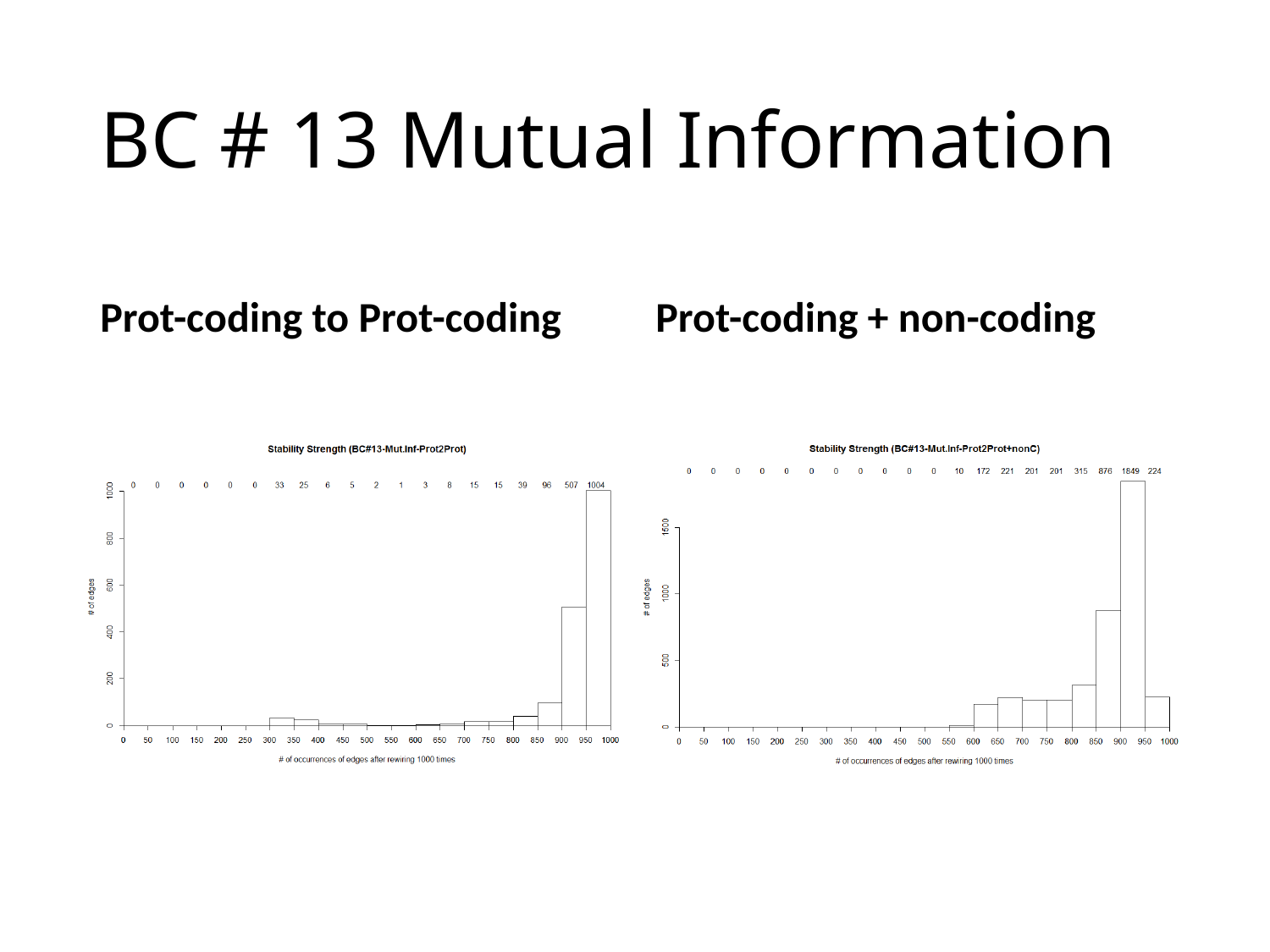

# BC # 13 Mutual Information
Prot-coding to Prot-coding
Prot-coding + non-coding

## Slide 10
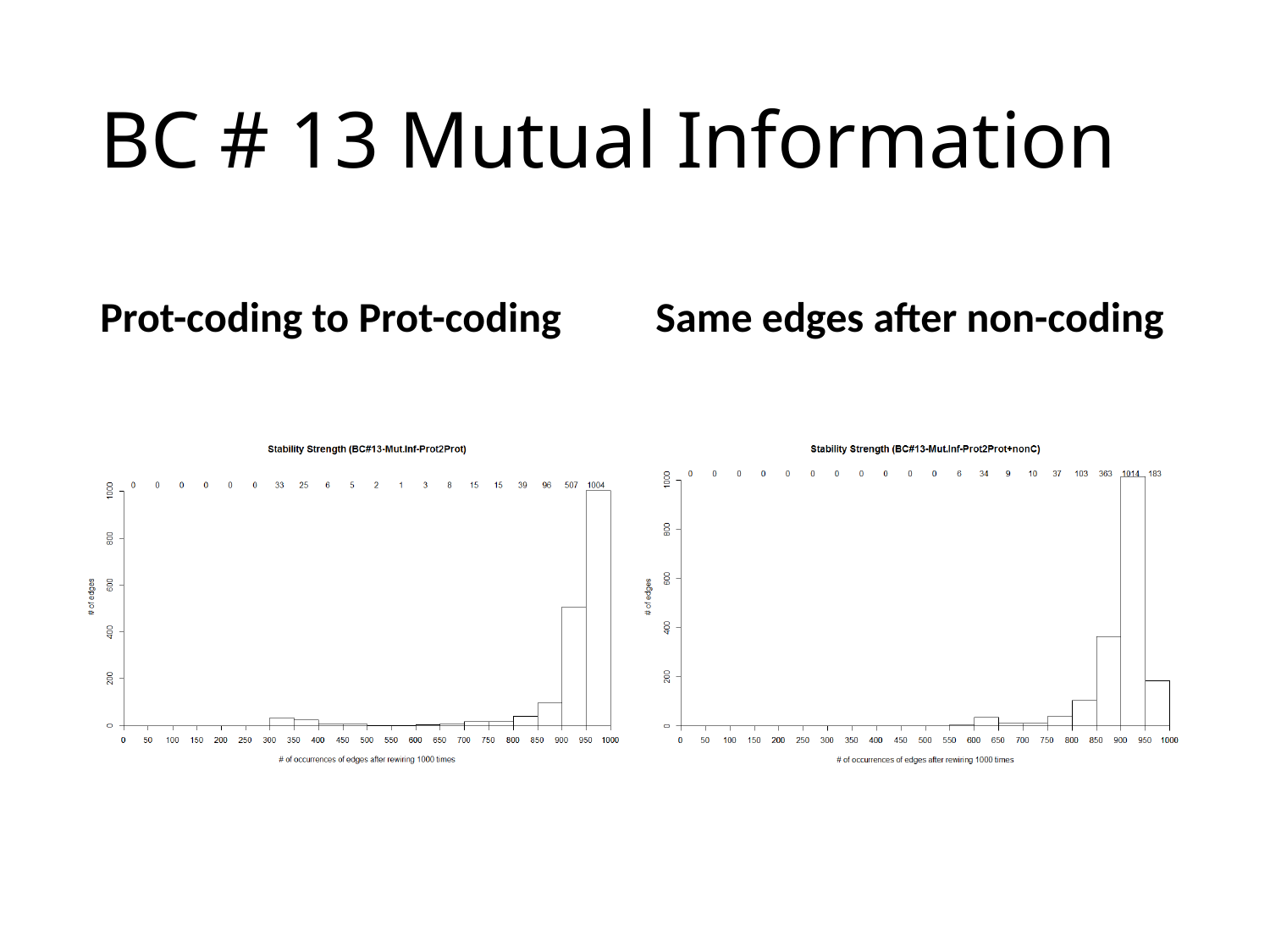

# BC # 13 Mutual Information
Prot-coding to Prot-coding
Same edges after non-coding

## Slide 11
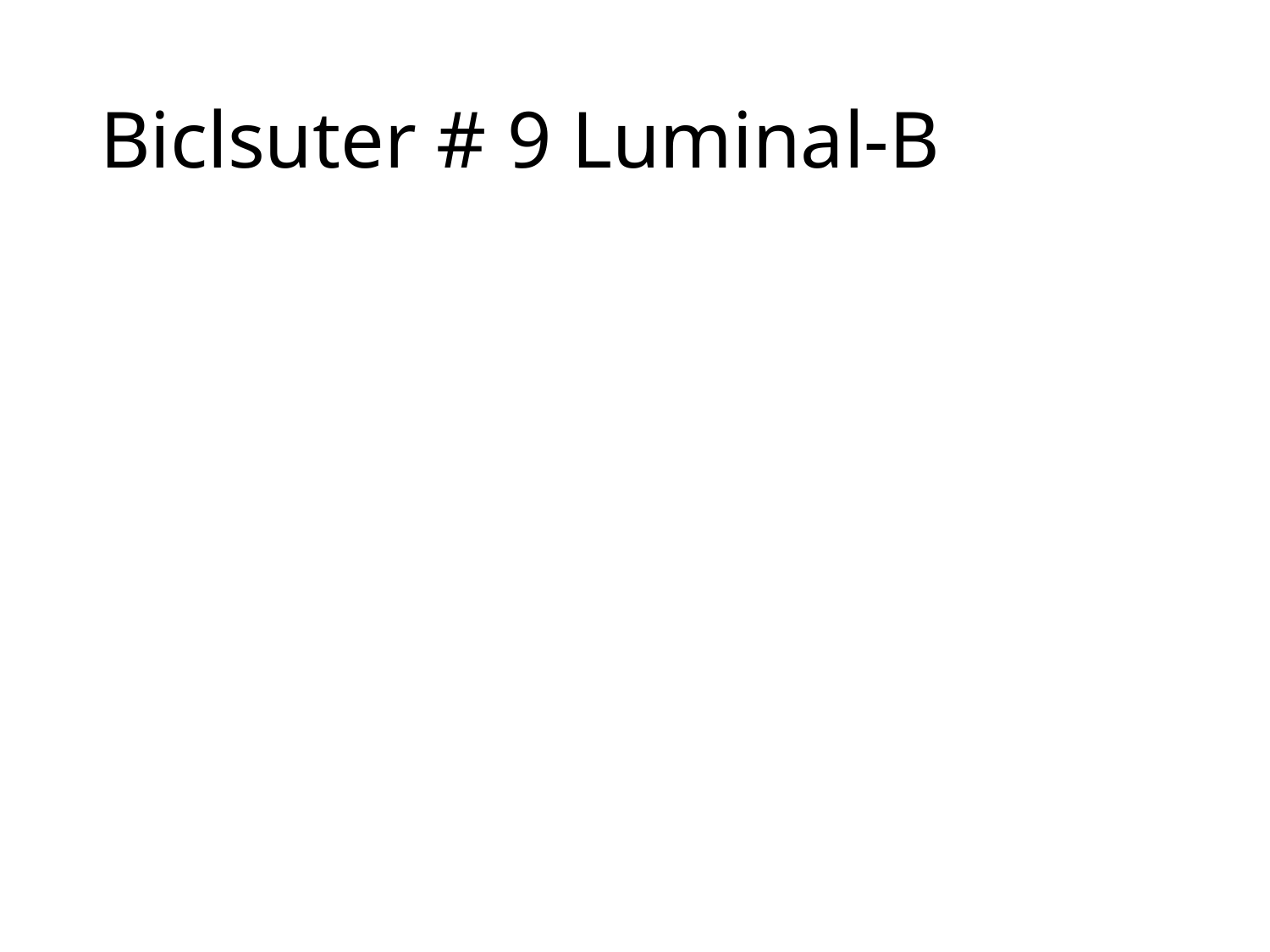

# Biclsuter # 9 Luminal-B

## Slide 12
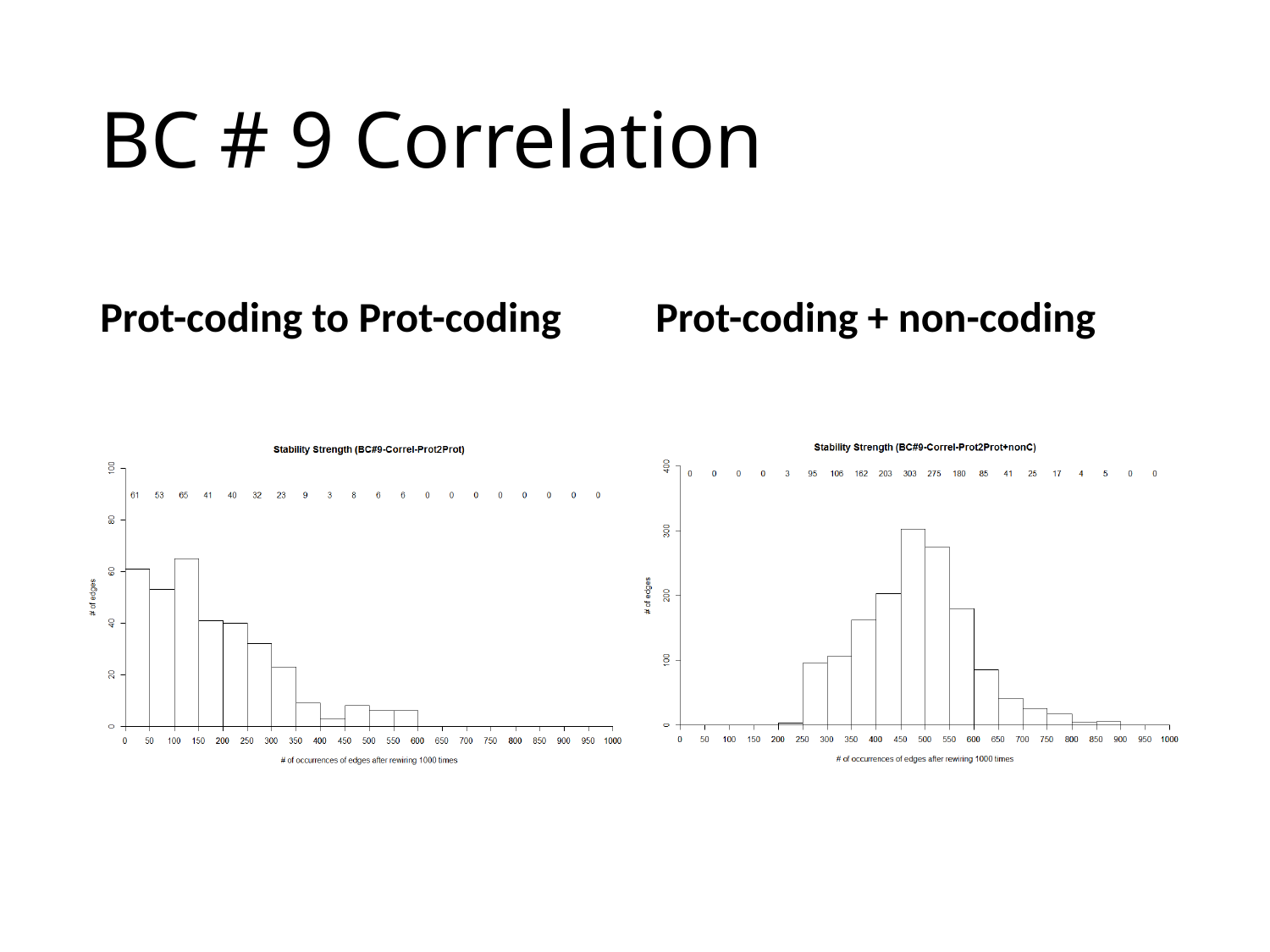

# BC # 9 Correlation
Prot-coding to Prot-coding
Prot-coding + non-coding

## Slide 13
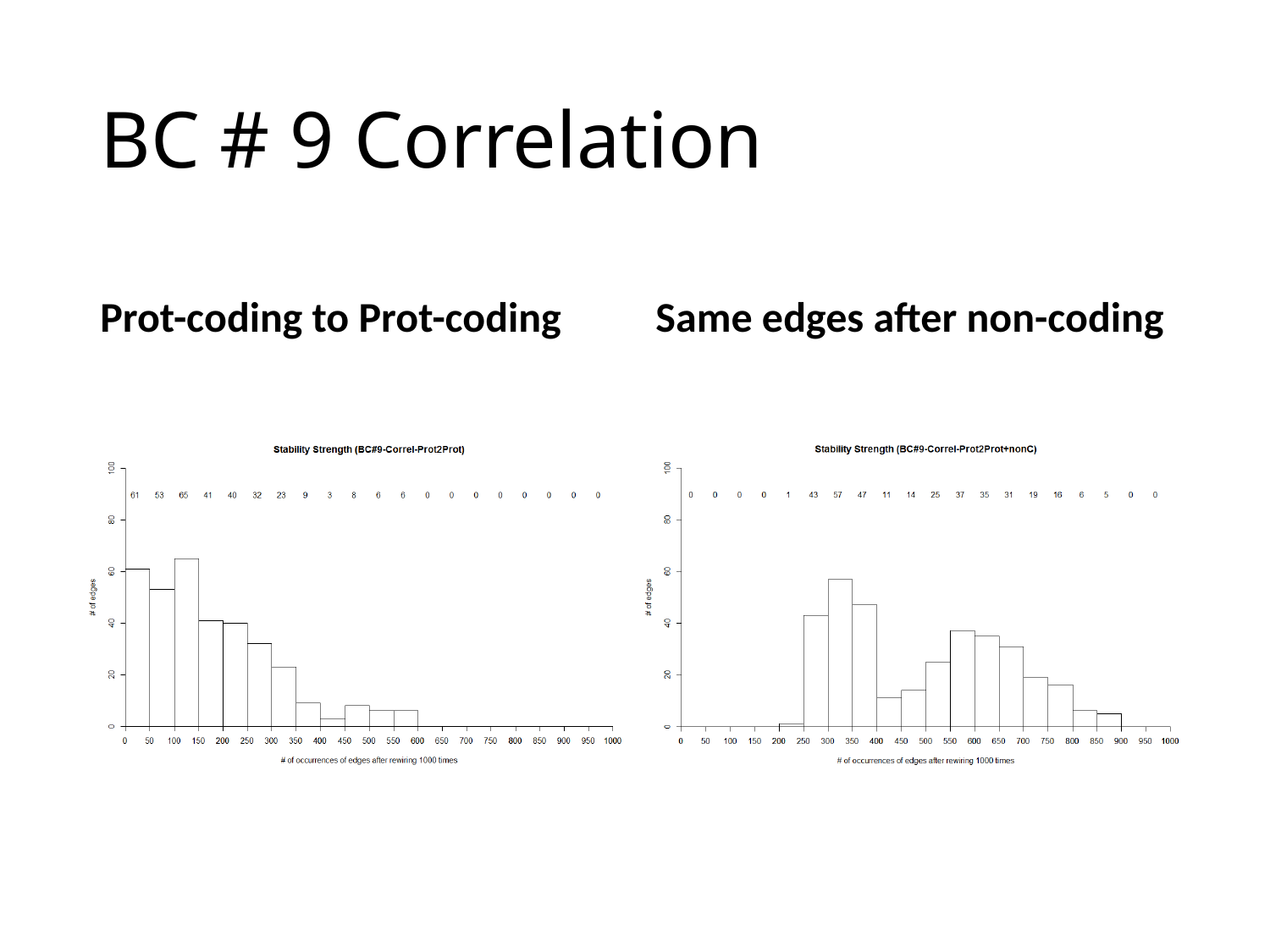

# BC # 9 Correlation
Prot-coding to Prot-coding
Same edges after non-coding

## Slide 14
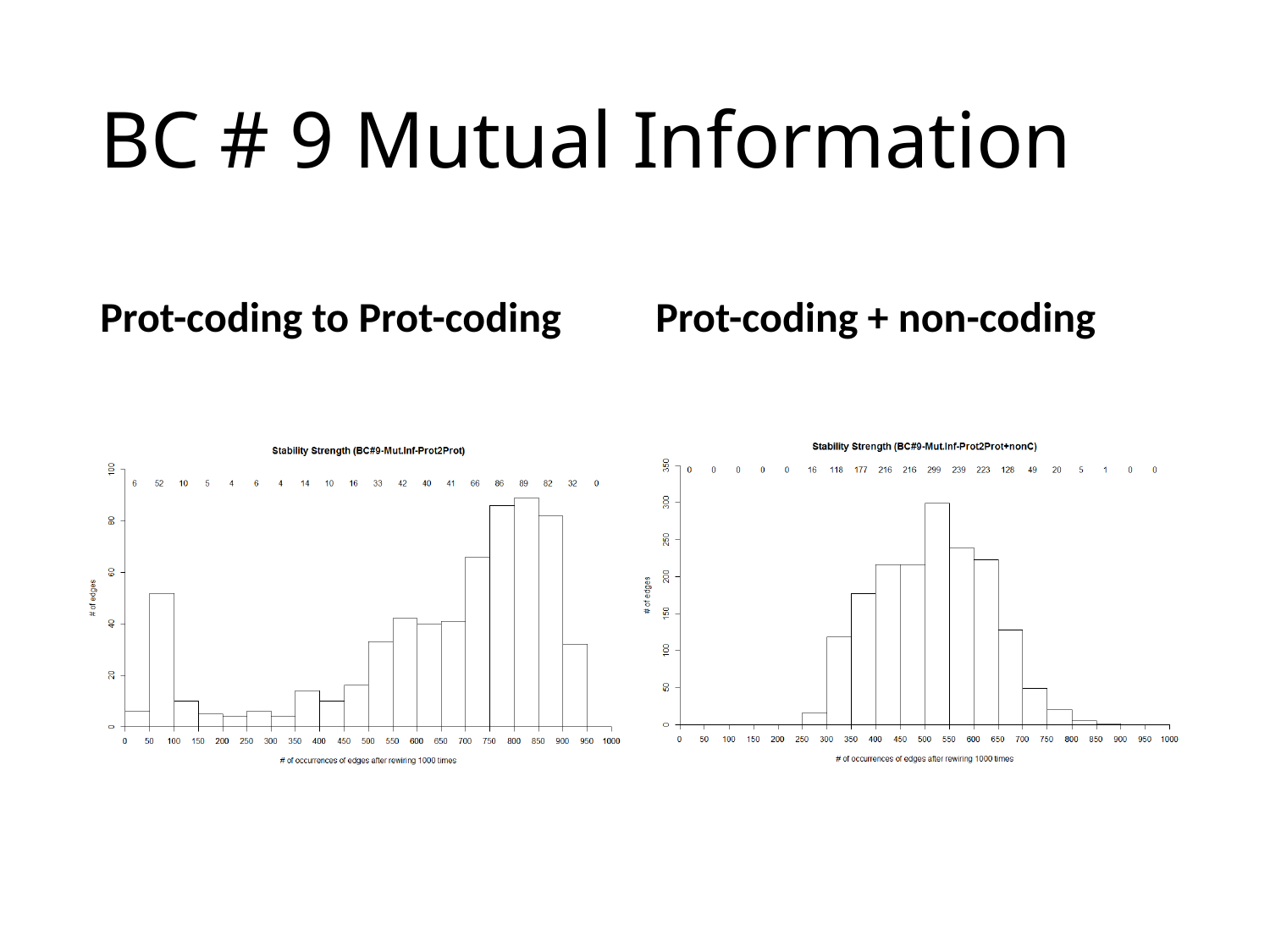

# BC # 9 Mutual Information
Prot-coding to Prot-coding
Prot-coding + non-coding

## Slide 15
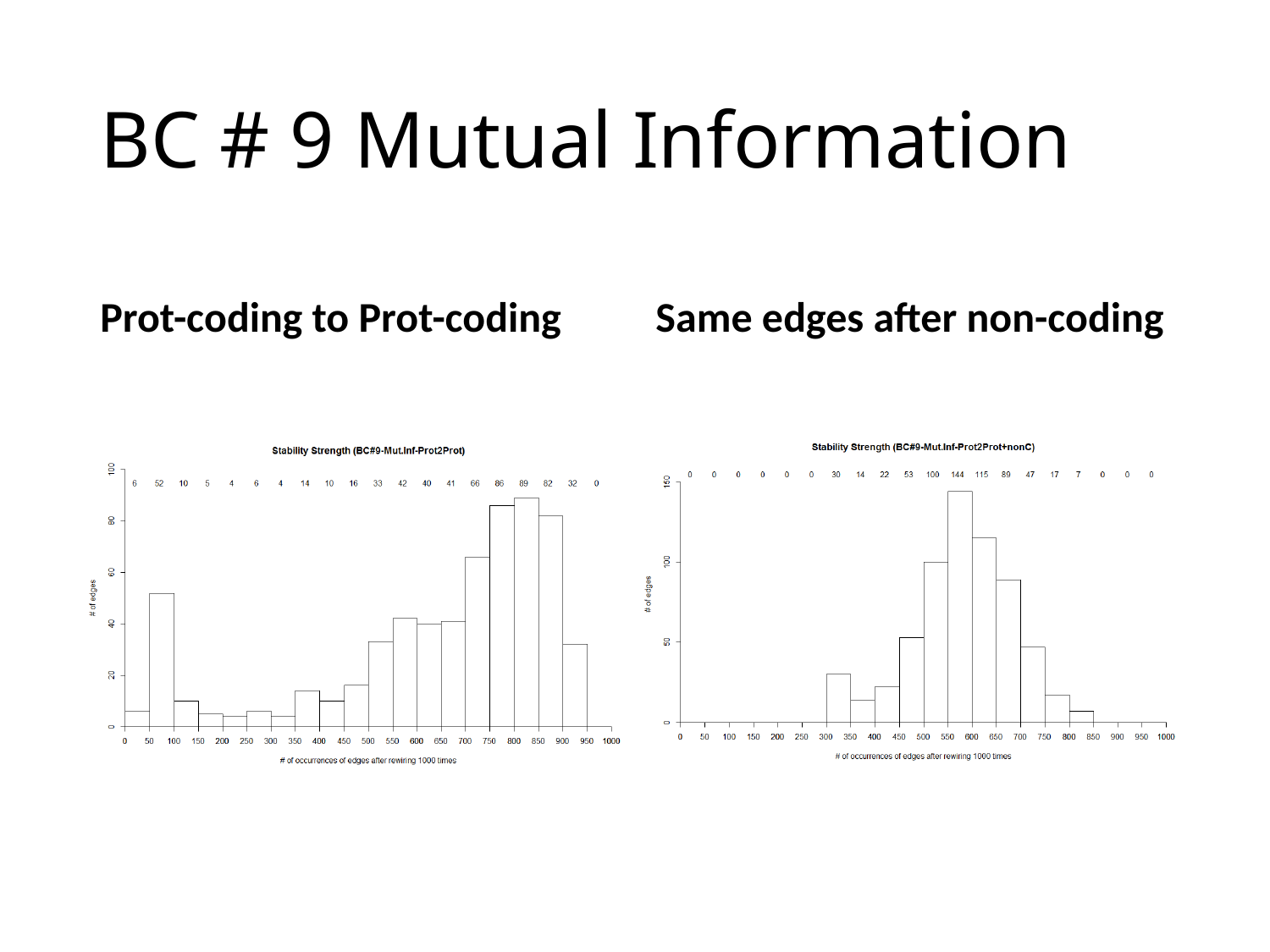

# BC # 9 Mutual Information
Prot-coding to Prot-coding
Same edges after non-coding
